# Simulating immunosuppressive mechanism of *Microplitis bicoloratus bracovirus* coordinately fights *Spodoptera frugiperda*

**DOI:** 10.3389/fimmu.2023.1289477

**Published:** 2023-12-11

**Authors:** Xing-Cheng Li, Yin-Chen Ma, Jin Long, Xiang Yan, Nan-Nan Peng, Cheng-Hui Cai, Wen-Feng Zhong, Yong-Biao Huang, Xin Qiao, Li-Xiang Zhou, Qiu-Chen Cai, Chang-Xu Cheng, Gui-Fang Zhou, Yun-Feng Han, Hong-Yu Liu, Qi Zhang, Hong-Mei Tang, Jiang-Hui Meng, Kai-Jun Luo

**Affiliations:** ^1^ School of Life Sciences, Yunnan University, Kunming, China; ^2^ Yunnan International Joint Laboratory of Virology & Immunology, Yunnan University, Kunming, China; ^3^ Key Laboratory of the University in Yunnan Province for International Cooperation in Intercellular Communications and Regulations, Yunnan University, Kunming, China

**Keywords:** bracovirus, transcription signaling pathway, translation signaling pathway, cell-cell communication, PGE_2_ pathway, dsRNA, *S. frugiperda*, ROS

## Abstract

Parasitoid wasps control pests via a precise attack leading to the death of the pest. However, parasitoid larvae exhibit self-protection strategies against bracovirus-induced reactive oxygen species impairment. This has a detrimental effect on pest control. Here, we report a strategy for simulating *Microplitis bicoloratus bracovirus* using *Mix-T* dsRNA targeting 14 genes associated with transcription, translation, cell–cell communication, and humoral signaling pathways in the host, and from wasp extracellular superoxide dismutases. We implemented either one-time feeding to the younger instar larvae or spraying once on the corn leaves, to effectively control the invading pest *Spodoptera frugiperda*. This highlights the conserved principle of “biological pest control,” as elucidated by the triple interaction of parasitoid-bracovirus-host in a cooperation strategy of bracovirus against its pest host.

## Graphical Abstract

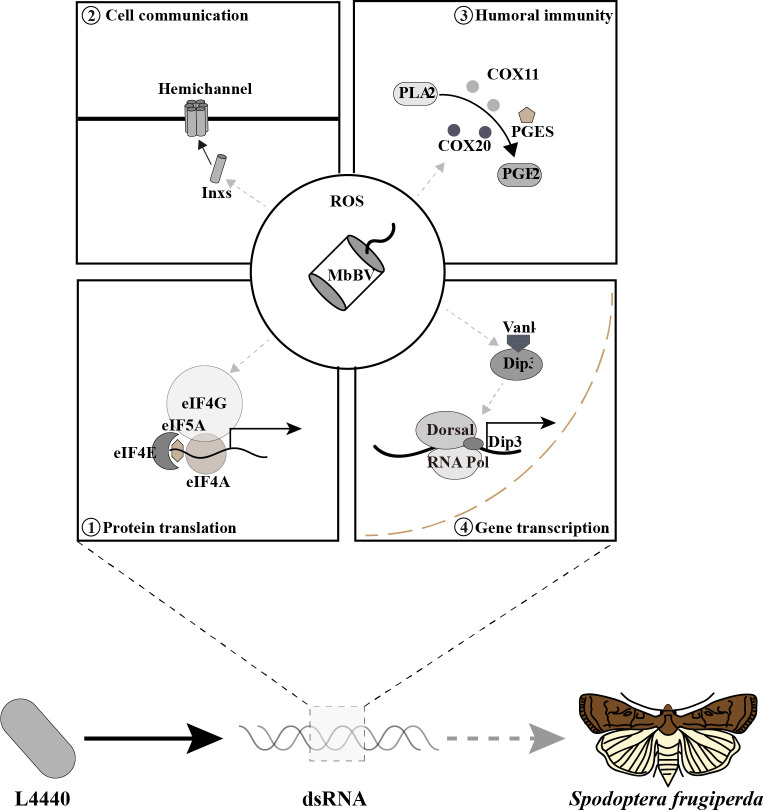

## Introduction

Symbiotic polydnaviruses do not replicate in host cells, making it is impossible to mass-produce them for use in pest control. Symbiotic polydnavirus in hymenopteran wasps suppresses lepidopteran host immune system to assist the development of parasitoids (1). However, the parasitoid also needs sufficient time to complete its development adapting to the increase in reactive oxygen species (ROS) levels triggered by the bracovirus (2). Bracovirus inhibits host immunity at three levels: a) integration into host DNA, directly damaging important host immune genes; b) the viral protein inhibiting transcription of host immune genes and c) translation of host transcriptome to suppress some of the main cellular signaling pathways (3–5). Meanwhile, the parasitoid needs resources from the host for its development (3, 4). These balances are established through interplay between the bracovirus and its symbiotic parasitoid, which complicates the development of strategies for the utility of bracovirus in biological control. Therefore, novel approaches are necessary to direct pest control in the field of biological control.

Indeed, host–parasitoid–bracovirus interactions are well-documented for individual genes or pathways during the process of parasitism. However, their coordinate involvement in the context of these triple interactions and their utility for biological control are not well understood. Although, the direct release of natural enemies is a traditional, conserved, biological strategy, the important aspect is to devise methods of increased efficiency to control agricultural pests using the pest–killing mechanisms of their natural enemies. In such tri-trophic interactions among bracovirus, host, and parasitoid wasps, the often ignored key question is the attack on the host by bracoviruses and parasitoid wasps in an impaired environment triggered by bracovirus for self-protection. The latter is a negative factor reducing the effectivity of biological control using the parasitoid, contrary to the expected quick killing of the pest. The bracovirus triggers the generation of ROS, which is degraded by the extracellular superoxide dismutases (ecSODs) from parasitoid larvae (2). Alteration of ROS homeostasis affects lifespan as shown previously in the yeast, *Saccharomyces cerevisiae* (6).

*Spodoptera frugiperda* (Lepidoptera, Noctuidae), an invasive globally polyphagous pest, is a non–native host of the wasp *Microplitis bicoloratus*, which in turn is the native parasitoid of *Spodoptera litura* (Lepidoptera, Noctuidae) and carries the symbiotic bracovirus (7). Even in laboratory, *M. bicoloratus* demonstrates less parasitism towards *S. frugiperda*, which is a migratory agricultural pest native to North and South America. It was first discovered in Africa (8) and subsequently spread to Europe and Asia (9), especially to Yunnan in China (10–12). The genomes of *S. litura* (13) and *S. frugiperda* (14) and its Sf9 cell line (15) have been sequenced; they have high sequence similarity and these organisms share common signaling pathways, including those involved in transcription, translation, cell communication, and humoral responses, based on our research using Sf9 cells (3, 4). Such similarity between the two hosts prompted us to harness effects of *Microplitis bicoloratus bracovirus* (MbBV) on host gene expression to control *S. frugiperda*.

Bracovirus integrates into the host genome and inhibits host immune responses through four main pathways: transcription, translation, humoral and cell communication. The viral ankyrin (Vank) proteins inhibit the dorsal interaction proteins 3 (Dip3) and thereby reduce the transcription of key immune factors, such as antimicrobial peptides, apoptotic factors, and eukaryotic translation factors (eIFs) (5, 16). Vank proteins also disrupt the activity of translation factors along the eIF4E-4A axis (1, 5) and the eIF5A–hypusine-related components deoxyhypusine synthase (DHYS) and deoxyhypusine hydroxylase (DOHH) (3), thereby causing protein reduction. Furthermore, it disrupts intracellular communication by closing hemichannels (formed by *Inx1*, *Inx2*, *Inx3*, and *Inx4*), promotes the disassembly of apoptotic bodies (4), and transmits immunosuppressive signaling (3). In addition, MbBV inhibits antimicrobial peptide expression, thus modulating the humoral (PGE_2_) pathway.

However, a simultaneous inhibition of the four major signaling pathways of *S. litura* by the bracovirus, which controls the congeneric pest, and the utilization of this mechanism has not been reported yet. We hypothesized that eliminating the negative effect of decreased ROS levels caused by parasitoid self-protection would fully simulate a bracoviral attack to control *S. frugiperda* by targeting four main signaling pathways, resulting in increased ROS levels and thus pest-killing. Recently, RNA interference (RNAi) technology has been widely used in pest management (17–20), although RNAi efficiency in lepidopteran species, especially *in vivo* is still controversial in the community (21, 22). Many studies have reported the use of dsRNA to down-regulate insect genes through feeding. Recently, dsRNA feeding was used for downregulating genes in insects, such as dsRNases of corn leafhopper, *Dalbulus maidis* (23) and the glutamate-gated chloride channel gene of the fall armyworm, *Spodoptera frugiperda* (24). Further, dsRNA is taken up through an active process involving receptor-mediated endocytosis in *Drosophila emanogster S2 cells* (25). In this study, we have tested how dsRNA can be specifically used to knock-down host genes targeted by bracovirus and to mimic the immunosuppression of *S. frugiperda* larvae. We used 14 *Mix-T* double-stranded RNA (dsRNA) to simulate bracovirus through one-time feeding and found that different pathways performed different functions against *S. frugiperda*, in coordination. We believe that our approach considers the tri-trophic interactions that enhance the effectiveness of the biological control of the pest. Furthermore, we introduce a novel perspective to develop biocontrol strategies.

## Results

### Simulating bracovirus by using *Mix-T* 14 dsRNAs against *S. frugiperda* through one-time feeding

**Figure 1:**
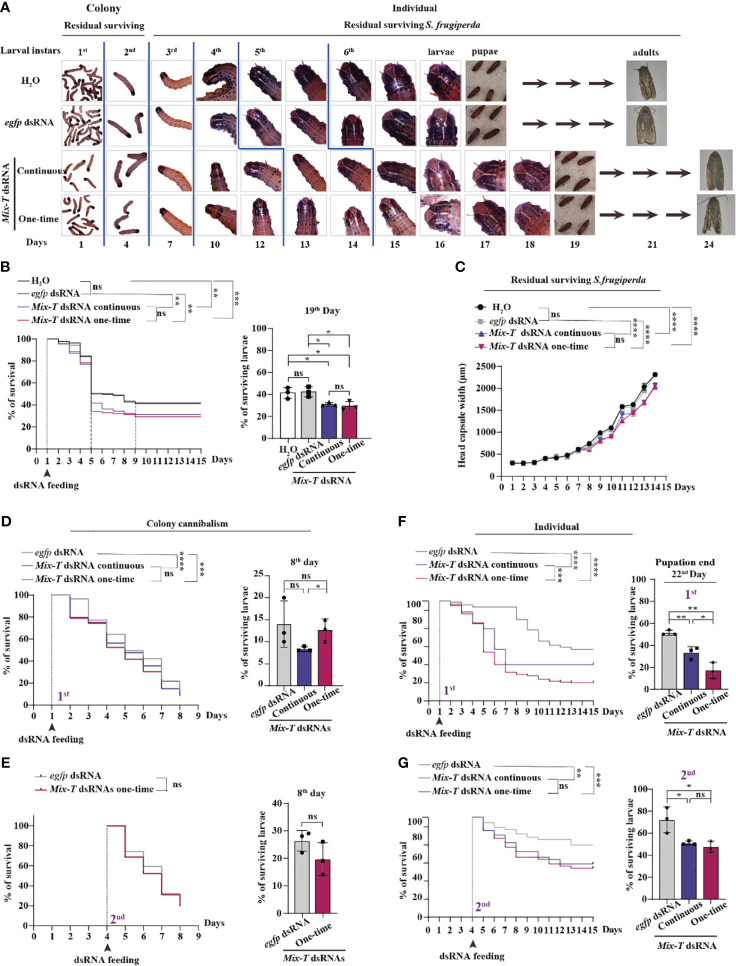
Simulating bracovirus against *S. frugiperda *via the established one-time feeding of *Mix-T* dsRNA. **(A)** The life cycle of *S. frugiperda* presented in the colony from the 1^st^ to 2^nd^ instar larvae and in individuals from the 3^rd^ to 6^th^ instar larvae after continuous and one-time feeding with *Mix-T* dsRNA. **(B)** Survival curve of *S. frugiperda* after feeding with *Mix-T* dsRNA from the 1^st^ to the 15^th^ day and survival rate on the 19^th^ day, at the end of pupation. ns, *p* (H_2_O: *egfp* dsRNA) = 0.8976; ** *p* (H_2_O: *Mix-T* dsRNA continuous) = 0.0022; *** *p* (H_2_O: *Mix-T* dsRNA one-time) = 0.0002; ** *p* (*egfp* dsRNA: *Mix-T* dsRNA continuous) = 0.0015; ** *p* (*egfp* dsRNA: *Mix-T* dsRNA one-time) = 0.0004; ns, *p* (*Mix-T* dsRNA continuous: *Mix-T* dsRNA one-time) = 0.7120. **(C)** The head capsule width of residual survival *S. frugiperda* after feeding with *Mix-T* dsRNA from the 1^st^ to the 15^th^ day. The head capsule width of residual survival was analyzed using two-way ANOVA. The head capsule width was compared using the Tukey’s multiple comparisons test. *F_0.05_* (3,112) = 50.30, *p* < 0.0001. ns, *p* (H_2_O: *egfp* dsRNA) = 0.1460; ****p (H_2_O: *Mix-T* dsRNA continuous) < 0.0001; ****p (H_2_O: *Mix-T* dsRNA one-time) < 0.0001; ****p (*egfp* dsRNA: *Mix-T* dsRNA continuous) < 0.0001; ****p (*egfp* dsRNA: *Mix-T* dsRNA one-time) < 0.0001; ns, *p* (*Mix-T* dsRNA continuous: *Mix-T* dsRNA one-time) = 0.3425. **(D)** Survival curve of colony cannibalism of 1^st^ instar *S. frugiperda* larvae after feeding with *Mix-T* dsRNA from the 1^st^ to the 8^th^ day and the survival rate on the 8^th^ day upon molting into the 3^rd^ instar. **** *p* (*egfp* dsRNA: *Mix-T* dsRNA continuous) < 0.0001; *** *p* (*egfp* dsRNA: *Mix-T* dsRNA one-time) = 0.0009; ns, *p* (*Mix-T* dsRNA continuous: *Mix-T* dsRNA one-time) = 0.6839. **(E)** Survival curve of the colony cannibalism of 2^nd^ instar *S. frugiperda* larvae after feeding with *Mix-T* dsRNA from the 4^th^ to the 8^th^ day and the survival rate at the 8^th^ day upon turning into 3^rd^ instar. ns, *p* (*egfp* dsRNA: *Mix-T* dsRNA one-time) = 0.0579. **(F)** Survival curve of individual 1^st^ instar *S. frugiperda* larvae after feeding with *Mix-T* dsRNA from the starting day to the 22^nd^ day and the survival rate on the 22^nd^ day at the end of pupation. **** *p* (*egfp* dsRNA: *Mix-T* dsRNA continuous) < 0.0001; **** *p* (*egfp* dsRNA: *Mix-T* dsRNA one-time) < 0.0001; *** *p* (*Mix-T* dsRNA continuous: *Mix-T* dsRNA one-time) < 0.0009. **(G)** Survival curve of individual 2^nd^ instar *S. frugiperda* larvae after feeding with *Mix-T* dsRNA from the starting day to the 22^nd^ day and the survival rate on the 22^nd^ day at the end of pupation. ** *p* (*egfp* dsRNA: *Mix-T* dsRNA continuous) = 0.0064; *** *p* (*egfp* dsRNA: *Mix-T* dsRNA one-time) = 0.0008; ns, *p* (*Mix-T* dsRNA continuous: *Mix-T* dsRNA one-time) = 0.5347. Survival curves were compared using the log-rank (Mantel–Cox) test [x^2^(3) = 22.09] in **(B)**, [x^2^(2) = 5.725] in **(D)**, [x^2^(2) = 63.23] in **(E)**, and [x^2^(2) = 11.71] in **(F)**. In all graphs, *p < 0.05, **p < 0.01, ***p < 0.001, ****p < 0.0001, ns, no significance; the error bars represent the SEM. Unpaired Student’s *t*-test with Holm–Sidak method for multiple *t* test; n = 3.

Based on the behavior of *S. frugiperda* in the field, we designed assays for larvae in colonies from 1^st^ to 2^nd^ and for individual larvae from 3^rd^ to 6^th^ instars, using continuous and one-time feeding methods (**Figure 1A**). The residual survival of 1^st^ to 3^rd^ instars showed consistent development in controls, H_2_O and *egfp* dsRNA, and *Mix-T* 14 dsRNA with both continuous and one-time feeding. We observed that the 4^th^ instar lasted two days longer (dsRNA feeding than two controls), 5^th^ instar, one day longer, 6^th^ instar, two days longer, and the pupation stage lasted one-day longer compared to controls (**Figure 1A**). The data showed that the residual surviving *S. frugiperda* larvae had a prolonged lifecycle, implying decreased generations per year.

The survival curves showed that the survival rates of both the dsRNA feeding groups were significantly lower than those of the controls; furthermore, there were no significant differences in both continuous and one-time dsRNA feeding (**Figure 1B**). These data suggest that, by only feeding one-time before the 4^th^ instar stage at 9 days, *Mix-T* 14 dsRNA kills pests with efficiency. From days 9—19, until the end of pupation, the survival percentage of larvae treated with *Mix-T* dsRNA was significantly lower than that of the control group; however, no significant differences were found upon comparing the two dsRNA treatments and control groups, implying that *Mix-T* dsRNA showed a sustained effect. Subsequently, the development of the residual surviving *S. frugiperda* larvae was analyzed based on their head capsule width (7, 26). The larvae from both the dsRNA treatment groups showed significantly smaller head capsules than those of the two control groups with no significant differences between continuous and one-time feeding (**Figure 1C**). These findings suggest that one-time feeding of *Mix-T* dsRNA kills *S. frugiperda* larvae in the initial instar stages and sustainably inhibits the development of the residual surviving *S. frugiperda* larvae, suggesting the utility of *Mix-T* dsRNA for biocontrol.

Cannibalism of younger 2^nd^ instar larvae in the colony feeding was observed, which was analyzed using the survival rate assay at 5 days (**Figure 1B**). To detect whether dsRNA treatment affects cannibalism of *S. frugiperda* larvae, the 1^st^ instar larval colonies were fed on dsRNA until 3^rd^ instar stage. Both the feeding methods of *Mix-T* dsRNA resulted in a significantly lower survival of larvae compared with the feeding of control *egfp* dsRNA (**Figure 1D**), suggesting that dsRNA promoted the cannibalism of lower-stage instars. By the 8^th^ day, dsRNA treatments and control group showed no significant differences, suggesting that *Mix-T* dsRNA has no sustained effect on promoting cannibalism beyond the 2^nd^ instar stage. Furthermore, the cannibalism of the colony’s 2^nd^ instar larvae was not significantly different across the treatment groups until the 3^rd^ instar (**Figure 1E**). The data confirmed that true cannibalism (aggressive attitude) of larvae, not an increase in appeal, occurs at the beginning of 1^st^ instar, implying that *Mix-T* dsRNA can be used against a newly hatching colony to elevate their cannibalism.

To further confirm the effect of one-time dsRNA feeding on lower instar larval individuals, 1^st^ instar larvae were separated. The percentage of survival for both dsRNA treatments was significantly lower compared with that of the control *egfp* dsRNA; furthermore, the percentage of survival with one-time *Mix-T* dsRNA feeding was significantly lower than with continuous feeding (**Figure 1F**) at the 22^nd^ day of pupation end. The number of larvae in *Mix-T* dsRNA-treated groups were significantly lower than in the control groups, suggesting that *Mix-T* dsRNA showed a persistent effect on the larvae. Similar results were obtained for the individual 2^nd^ instars indicating that one-time feeding could kill larvae starting from 2^nd^ instar stage (**Figure 1G**). However, this was not the case with the individual 3^rd^ (**Figure S1A**), 4^th^ (**Figure S1B**), 5^th^ (**Figure S1C**) and 6^th^ (**Figure S1D**) instar larvae, suggesting that *Mix-T* 14 dsRNA can be used against *S. frugiperda* during initial instar larval stages.

Taken together, these findings showed that simulating bracovirus using dsRNA kills *S. frugiperda* from the lower 3^rd^ instar stage onwards, inhibits the development of residual surviving *S. frugiperda* larvae and decreases the progression of generations, emphasizing that 14 *Mix-T* dsRNA can simulate bracovirus at least partially.

### *Mix-T* dsRNA transiently inhibits four major signaling pathways and increases cellular ROS levels

**Figure 2:**
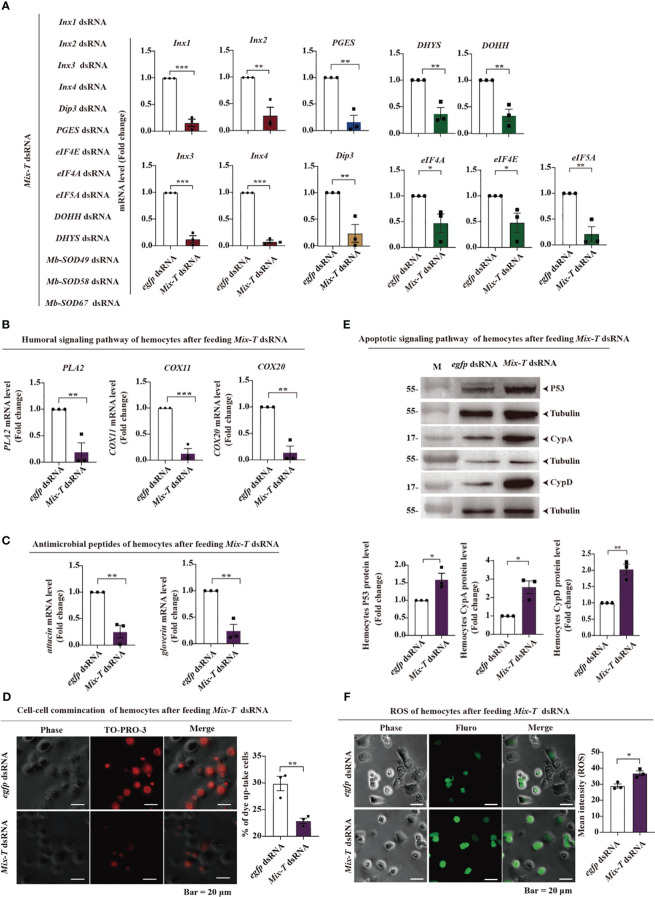
*Mix-T* dsRNA transiently inhibits four major signaling pathways and increases ROS levels. **(A–C)** RT-qPCR detection. RNAi silencing of 11 genes in four major pathways **(A)**; *PLA2*, *COX11* and *COX20*, which are associated with the humoral pathway **(B)**; *attacin* and *gloverin*, which encode antimicrobial peptides **(C)**. **(D)** Hemichannel detection of cellular communication using TO-PRO3 fluorescence dye. Scale bar, 20 µm. **(E)** The protein expression levels of p53, CypA, and CypD were detected by western blotting. **(F)** ROS detection in hemocytes. Scale bar, 20 µm. In all graphs, *p < 0.05, ∗∗*p* < 0.01, ***p < 0.001, ∗∗∗∗*p* < 0.0001, ns, no significance; the error bars represent the SEM. Unpaired Student’s *t*-test with Holm–Sidak method for multiple *t* test; n = 3.

To verify the molecular mechanisms of *Mix-T* dsRNA, we first assessed if the mRNA of the 14 genes were indeed silenced after feeding. Based on parasitoid-bracovirus-host interaction, parasitoid larvae require 6 days to complete development in the host hemocoel, and release ecSODs to reduce ROS triggered by MbBV infection throughout the developmental period (2, 7). The relationship between ROS and longevity has been reported in *Caenorhabditis elegans* (27). *Mix-T* dsRNA silenced 11 related genes involved in four main pathways mentioned above and three genes involved in neutralizing ROS from the host hemocytes (**Figure 2A**). Genes encoding humoral signaling molecules, *PLA2*, *COX11*, and *COX20* (**Figure 2B**), and those encoding antimicrobial peptides, *attacin* and *gloverin*, showed decreased expression (**Figure 2C**); cellular communication was also inhibited, as deduced by measuring the TO-PRO3 dye uptake (**Figure 2D**). In contrast, the expression of apoptosis-related proteins, p53, CypA, and CypD (**Figure 2E**), as well as the ROS generation, significantly increased (**Figure 2F**). These data suggested that four pathways were transiently suppressed, while cell apoptosis and ROS increased to create an impaired environment in the host, implying that *Mix-T* dsRNA simulates bracovirus and kills *S. frugiperda* by modulating these pathways.

### Cooperative functioning of *Dip3*, *eIFs*, *PCCPs* dsRNAs kills *S. frugiperda* in the feeding stage

**Figure 3:**
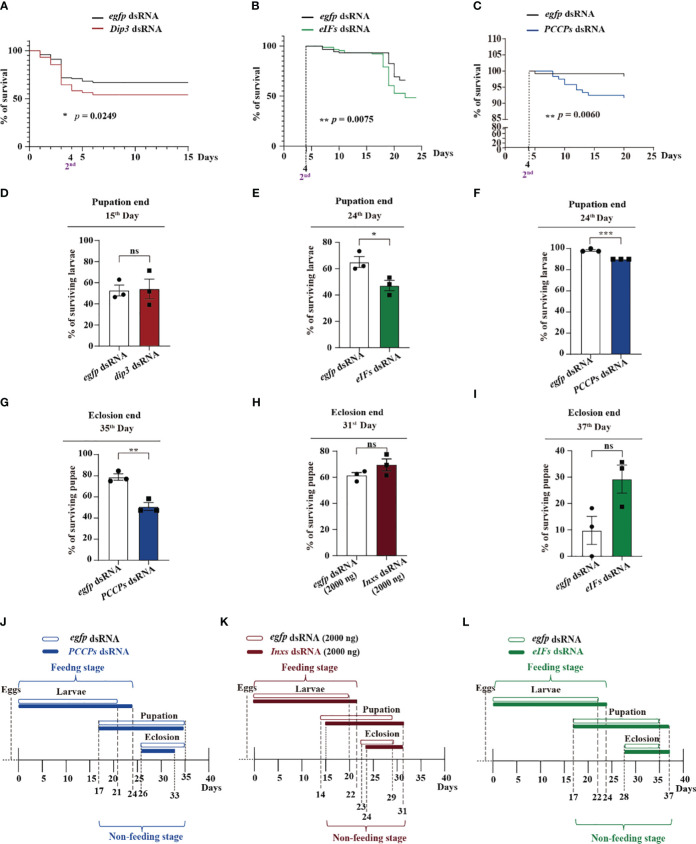
*Dip3*, *eIFs*, *Inxs*, and *PCCPs* dsRNAs co-operatively function against *S. frugiperda* at the feeding and non-feeding stages. **(A–C)** The survival curve of *S. frugiperda* after feeding with *Dip3* **(A)**, *eIFs* **(B)**, and *PCCPs* **(C)** dsRNA. **(D–F)** Survival rates of *S. frugiperda* larvae after feeding with *Dip3* **(D)**, *eIFs* **(E)**, and *PCCPs* **(F)** dsRNA at the end of pupation. **(G–I)** The *S. frugiperda* pupae that survived after feeding with *PCCPs* **(G)**, *Inxs* **(H)**, and *eIFs* **(I)** dsRNA at the end of eclosion. **(J–L)** Time of feeding and non-feeding (pupation and eclosion) stages of *S. frugiperda* after feeding with *PCCPs* **(D)**, *Inxs* **(E)**, and *eIFs* **(F)** dsRNA. Survival curves were compared using the log-rank (Mantel–Cox) test [x^2^(1) = 5.029] in **(A)**, [x^2^(1) = 7.142] in **(B)**, and [x^2^(1) = 7.546] in **(C)**. In all graphs, *p < 0.05, **p < 0.01, ***p < 0.001, ns, no significance; the error bars represent the SEM. Unpaired Student’s *t*-test with Holm–Sidak method for multiple *t* test; n = 3.

Administration of dsRNA against the transcriptional co-factor *Dip3* quickly and significantly killed young larvae (**Figure 3A, D**), although it failed to kill larvae over 2^nd^ instar stage. On the 15^th^ day at the end of pupation, the surviving larvae from the *Dip3* dsRNA group showed no significant differences compared with the control *egfp* dsRNA group, implying that *Dip3* dsRNA is an efficient pesticide without sustained effects on the larva (**Figure 3D**). From 2^nd^ instar to the final larval stage, the percentage of surviving larvae from the *eIFs* dsRNA group was significantly lower than those from the control *egfp* dsRNA group (**Figure 3B, E**). Subsequently, at the end of pupation, the number of surviving larvae treated with *eIFs* dsRNA was significantly lower compared with that of the control group, implying that *eIFs* dsRNA sustainably kill different instar larvae until the end of pupation (**Figure 3E**). Similar results were obtained upon treatment with *PCCPs* dsRNA (**Figure 3C, F**). In contrast, *Inxs* dsRNA administration did not kill the larvae and showed no effect on the pupae (**Figure S2A, B**). These data suggest the complementary function of these three main pathways to kill *S. frugiperda* in the feeding stage, and that they are potentially modulated by each other.

### *PCCPs, eIFs*, and *Inxs* dsRNAs cooperatively function against *S. frugiperda* larvae in the non-feeding stage

Killing pest larvae in the non-feeding stage is also a strategy of biological control. At the end of eclosion, *PCCPs* dsRNA were observed to kill pupae significantly in the non-feeding stage (**Figure 3G**), while the downregulation of genes involved in the other three pathways namely though, *Inxs* dsRNA (**Figure 3H**), *eIFs* dsRNA (**Figure 3I**), and *Dip3* dsRNA (**Figure S2C**), did not kill pupae in the non-feeding stage. Interestingly, *PCCPs* dsRNAs (**Figure 3J**) and *Dips* dsRNA (**Figure S2D**) did not increase the developmental time; while *Inxs* dsRNA (**Figure 3K**) and *eIFs* dsRNA (**Figure 3L**) increased. These data suggest that these three main pathways, the humoral signaling, cellular communication, and the translation pathways, function cooperatively against the *S. frugiperda* larvae in its non-feeding stage.

### *eIFs*, *Inxs*, and *PCCP* dsRNAs cause immunosuppression in the residual surviving *S. frugiperda* larvae

**Figure 4:**
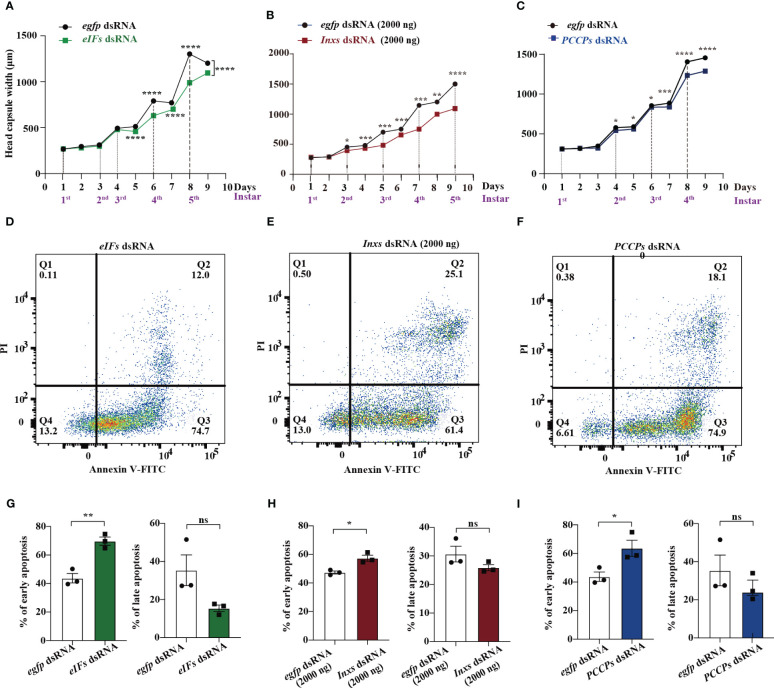
*eIFs*, *Inxs*, and *PCCP* dsRNAs cause immunosuppression in the residual surviving *S. frugiperda*. **(A–C)** The head capsule width of residual *S. frugiperda* larvae that survived after feeding with *eIFs* **(A)**, *Inxs* **(D)**, and *PCCPs* **(G)** dsRNA individually within 1–9 days. **(D–I)** Flow cytometry analysis of apoptotic hemocytes from residual *S. frugiperda* larvae that survived after feeding with *eIFs* **(D, G)**, *Inxs* **(E, H)**, and *PCCPs* **(F, I)** dsRNA, individually, within 1–9 days. In all graphs, *p < 0.05, **p < 0.01, ***p < 0.001, ****p < 0.0001, ns, no significance; the error bars represent the SEM. Unpaired Student’s *t*-test with Holm–Sidak method for multiple *t* test; n = 3.

Residual surviving *S. frugiperda* larvae were used for evaluating immunosuppression. Head capsule width and hemocyte apoptosis are hallmarks of immunosuppression mediated by the parasitization of *M. bicoloratus* (7, 28). The head capsule width of larvae from the *eIFs* dsRNA treatment group was significantly decreased from 5^th^ to 9^th^ day after continuous feeding (**Figure 4A** and **Figure S3A**). Meanwhile, early apoptosis significantly increased, as measured by flowcytometry (**Figure 4D, G**). The suppression of intracellular communication pathways resulted in significant immunosuppression in residual surviving *S. frugiperda* larvae, alongside significantly decreased head capsule width (**Figure 4B** and **Figure S3B**) and increased early apoptosis (**Figure 4E, H**). Similar results were found in the PCCP signaling pathway; the head capsule width of larvae feeding on *PCCPs* dsRNA was significantly decreased (**Figure 4C** and **Figure S3C**) and early apoptosis increased (**Figure 4F, I**). In contrast, administration of the dsRNA targeting the transcription cofactor *Dip3* (*Dip3* dsRNA) did not inhibit the immune response of the residual surviving larvae, determined based on the lack of effect on the head capsule (**Figure S3D**) and significantly higher early apoptosis (**Figure S3E, F**). These data suggest the common immunosuppressive functions of these three main pathways to cooperatively target the survival of the residual *S. frugiperda* larvae.

### *Mb-ecSODs* dsRNA enhances *Mix-T* dsRNA against *S. frugiperda* larvae

**Figure 5:**
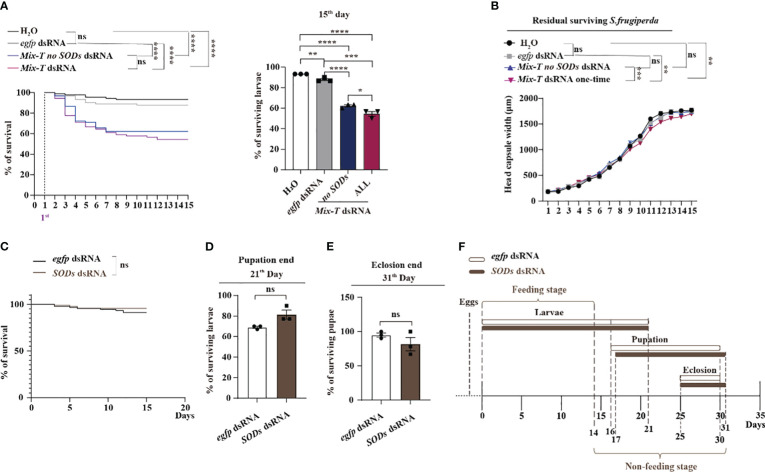
*Mb-ecSODs* dsRNA enhances *Mix-T* dsRNA against *S. frugiperda* larvae **(A)** Survival curve and survival rate of *S. frugiperda* fed with Mix-T no SODs and Mix-T dsRNA from day 1 to 15; ns, *p* (H_2_O: *egfp* dsRNA) = 0.1998; **** *p* (H_2_O: *Mix-T no SODs* dsRNA) < 0.0001; **** *p* (H_2_O: *Mix-T* dsRNA) < 0.0001; **** *p* (*egfp* dsRNA: *Mix-T no SODs* dsRNA) < 0.0001; **** *p* (*egfp* dsRNA: *Mix-T* dsRNA) <0.0001; ns, *p* (*Mix-T no sods* dsRNA: *Mix-T* dsRNA) = 0.2870. **(B)** The head capsule width of residual survival *S. frugiperda* after feeding with *Mix-T no SODs* dsRNA and *Mix-T* dsRNA from the 1^st^ to the 15^th^ day. The head capsule width of residual survival was analyzed using two-way ANOVA. The head capsule width was compared using the Tukey’s multiple comparisons test. *F*_0.05_ (3,120) = 7.868, *p* < 0.0001. ns, *p* (H_2_O: *egfp* dsRNA) = 0.9530; ns, *p* (H_2_O: *Mix-T no SODs* dsRNA) = 0.9095; ** *p* (H_2_O: *Mix-T* dsRNA) = 0.0013; ns, *p* (*egfp* dsRNA: *Mix-T no SODs* dsRNA) = 0.6337; ** *p* (*egfp* dsRNA: *Mix-T* dsRNA) = 0.0075; *** *p* (*Mix-T no SODs* dsRNA: *Mix-T* dsRNA) = 0.0001. **(C)** Survival curves and survival rate of *S. frugiperda* fed with *SODs* dsRNA and *egfp* dsRNA from day 1 to 15; ns, *p* (*egfp* dsRNA: *SODs* dsRNA) = 0.2396; **(D)** Survival rates of *S. frugiperda* larvae after feeding with *SODs* dsRNA at the end of pupation. **(E)** The *S. frugiperda* pupae that survived after feeding with *SODs* dsRNA at the end of eclosion. **(F)** Time of feeding and non-feeding (pupation and eclosion) stages of *S. frugiperda* after feeding with *SODs* dsRNA. Survival curves were compared using the log-rank (Mantel–Cox) test [x^2^(3) = 52.38] in **(A)**, and [x^2^(1) = 1.383] in **(C)**. In all graphs, *p < 0.05, **p < 0.01, ***p < 0.001, **** *p* < 0.0001, ns, no significance; the error bars represent the SEM. Unpaired Student’s *t*-test with Holm–Sidak method for multiple *t* test; n = 3.

Next, we investigated whether *M. bicoloratus ecSODs* dsRNA can enhance the effectiveness of *Mix-T* dsRNA against *S. frugiperda* in. Previous results showed that MbBV trigger the increase of ROS, which are reduced by *M. bicoloratus* ecSODs. Since the 14 *Mix-T* dsRNA increased ROS (**Figure 2F**), we wondered whether the addition of *ecSOD* dsRNA can enhance the death of *S. frugiperda*. When *Mix-T no SODs* dsRNA and *Mix-T* dsRNA were administered and the effects compared, the survival curves of the two dsRNA feeding groups were significantly lower than that the control groups. There were no significant differences between the two control groups and between the two dsRNA feeding groups, but the surviving larvae following treatment with *Mix-T no SODs* dsRNA were significantly higher than those after *Mix-T* dsRNA feeding at the 15^th^ days (**Figure 5A**). These results suggested that the added *ecSOD* dsRNA enhanced the death of *S. frugiperda*, at least partially. The development of the residual surviving *S. frugiperda* larvae was analyzed based on capsule width. The larvae from both the dsRNA treatment groups showed significantly smaller head capsule widths than those from the two control groups. Furthermore, there were significant differences between the *Mix-T no SODs* dsRNA and *Mix-T* dsRNA groups and both control groups (**Figure 5B**). These results imply that added *SODs* dsRNA inhibited the growth of *S. frugiperda* in larval stages and the development of residual larvae. Meanwhile, as per the parallel assays, the survival curves were not significantly different for the only single *SODs* dsRNA compared with control (**Figure 5C**). Moreover, there was no effect on the survival larval and pupal stages (**Figure 5D, E**). Furthermore, the developmental stage of larvae from both the treatments showed no significant differences (**Figure 5F**), implying that single *M. bicoloratus ecSODs* dsRNA has no effect on the *S. frugiperda*. The results supported our hypothesis that the addition of *M. bicoloratus SODs* dsRNA enhanced the effect of *Mix-T* dsRNA against *S. frugiperda*.

### *Mix-T* dsRNA one-time spray can effectively control *S. frugiperda* in the field

**Figure 6:**
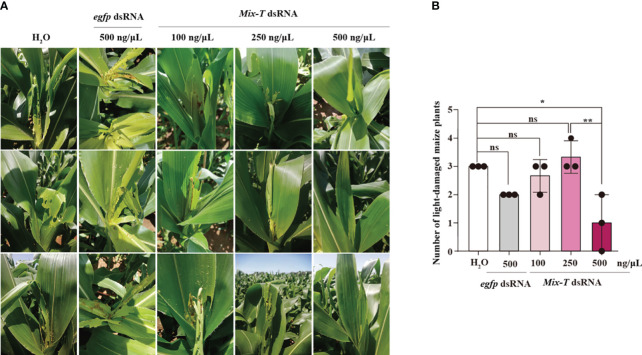
*Mix-T* dsRNA can effectively control *S. frugiperda* in the field **(A, B)** *Mix-T* dsRNAs showed dose-dependent effects against *S. frugiperda* in the field. *p < 0.05, **p < 0.01, ns, no significance; the error bars represent the SEM. David’s scale was used for statistics and unpaired Student’s *t*-test with Holm–Sidak method for multiple *t* test, n = 3.

To test the effectivity of *Mix-T* dsRNA to control *S. frugiperda* in the field, we performed spray assays on corn. Different instars with the same number of *S. frugiperda* were put on the heart leaves of corn, and different concentrations of *Mix-T* dsRNA were sprayed on all the leaves. Five days later, investigation of the corn field showed that, compared with the control groups, H_2_O and *egfp* dsRNA, the spraying of *Mix-T* dsRNA could effectively relieve pests, and the protective effect of *Mix-T* dsRNA on corn was dose-dependent (**Figure 6A**). The disaster situation of each maize was graded according to Davis survey method (**Figure S4**), and further statistical analysis showed that spraying 500 ng/µL *Mix-T* dsRNA could significantly control the pests. However, administration of other concentrations showed no significant difference compared with that of H_2_O. The number of plants with lower leaf damage after spraying 250 ng/µL dsRNA mixture was significantly less than that of the control group, i.e., only one-time spraying of 500 ng/µL dsRNA mixture could effectively control *S. frugiperda* (**Figure 6B**). This data suggests that *Mix-T* dsRNA simulated bracovirus can be a highly efficient pesticide to control *S. frugiperda*.

## Discussion

In the present study, we propose that simulating bracoviruses that are symbiotic with wasp by “one-time sting”, reduce pests with high efficiency and accuracy, and is a potential bio control strategy. Here, we used *Mix-T* dsRNA to target 14 genes, thereby simulating the wasp one-time sting to control *S. frugiperda* efficiency and accuracy by transiently suppressing four key pathways and increasing ROS. Eleven genes targeting four main pathways modulated by bracovirus and three genes from parasitoid larvae against ROS triggered by bracovirus have been used. Working in coordination, *Dip3*, *eIFs*, and *PCCPs* dsRNAs kill young instar larvae; *eIFs*, *Inxs*, and *PCCP* dsRNAs cause immunosuppression in the residual surviving *S. frugiperda* larvae; *eIFs*, *Inxs*, and *PCCP* dsRNAs function against *S. frugiperda* in its non-feeding stage. Collectively, our findings indicate that simulation of a bracoviral attack by using dsRNA is a promising biocontrol strategy.

*Mix-T* dsRNA instantly suppresses four main signaling pathways and eliminates SOD, thereby creating an impaired environment with higher apoptosis and ROS, which is a strategy of bracoviruses. Previous studies have also shown that appropriate ROS can prolong the lifespan of nematodes by inducing methylation of H3K4 to improve REDOX homeostasis in nematodes (29). However, research has shown that ROS imbalances lead to a decrease in longevity and cause disease in yeast and mice (30, 31). In the yeast, *Saccharomyces cerevisiae*, alteration of ROS homeostasis decreased its lifespan (30), implying that ROS is involved in its life-cycle regulation. These strategies appear to be utilized by bracovirus in the multi-trophic interactions of the polydnavirus-parasitoid-host. Moreover, ROS generation has been ignored in bio control tool development. *M. bicoloratus* parasitoid larvae secrete three proteins to reduce SOD and maintain lower ROS levels.

The simulation of bracoviral attack using one-time feeding dsRNA functions against lower instar *S. frugiperda* larvae*. S. frugiperda* larvae live in colonies during 1^st^-2^nd^ instars, and separate when the larvae molt into 3^rd^ instar. We used these behaviors to design the feeding mixture of 14 dsRNAs. One-time feeding of *Mix-T* dsRNA shows a high efficiency against *S. frugiperda* by killing larvae, inhibiting development of residual surviving larvae and prolonging the life cycle. Surprisingly, dsRNAs further triggered the 1^st^ instar larval cannibalism in the colony commonly found in the lepidoptera. This is the first report describing an additional role of dsRNA-mediated gene silencing, beyond the known general effects. This is similar to imidacloprid and acetamiprid, which can affect neurotransmitter transmission and nerve conduction in insects (5, 32). An interesting research found that starved male cells consume their own proteins (33). Serotonergic neurons in the brain of larval *S. frugiperda* are involved in multiple effects in a variety of behaviors, like feeding-related modulation (34). The stress caused by dsRNA administration may have triggered the nervous system to promote this cannibalism; excluding the cannibalism of 1^st^ instar, one-time feeding kills individual larva in 1^st^ and 2^nd^ instar. Importantly, *Mix-T* dsRNA works against *S. frugiperda* in different stages in various ways, highlighting the relevance of these strategies based on bracovirus-wasp-host interactions. The efficiency of simulation by RNAi technique can be further improved, and the technique can then be used in biological control. Similar to RNAi silencing, lepidopteran insects perform substance exchange and immune functions through the circulation of hemolymph in the body, giving dsRNA a chance to pass through the insect gut to the hemolymph and to be delivered into the cell to perform its functions (35–37). Needless to say, targeting key genes for growth and development is better at eliminating pests. Therefore, the genes selected in this study are those that have been proven to affect larval development and immunity and were based on the immunosuppressive mechanism of insect parasitism. In the parasitic system, the significant downregulation of these genes resulted in growth retardation and immunosuppression of larvae (38). Collectively considering all functions of 14 *Mix-T* dsRNA, in the fields, one-time spraying reduced the damage of maize plant.

Four main pathways, transcription, translation, PGE_2_ regulation, and intracellular communication, play different roles against *S. frugiperda* during both the feeding and non-feeding stages, and their cooperative interactions improve the efficiency of our method. Three pathways blocked by dsRNA individually, directly kill lower instar *S. frugiperda*. The most important target of the bracovirus is the transcriptional pathway as evidenced by *Dip3* dsRNA killings *S. frugiperda* lower instar quickly. This is because Dip3, as a transcription factor, regulates the expression of multiple immune-phase genes. For example, Dip3 regulates the expression of eIF4E through the NF-κB pathway (5). Interestingly, *Dip3* dsRNA-mediated pest-killing has not been reported yet. Dip3 regulates transcription of genes, involved in survival. This effect is fast as well as short as seen only in lower 2^nd^ instar stage and has no effect on the residual larvae that survived. Fortunately, *eIFs* dsRNA and *PCCPs* dsRNA kill larva over 2^nd^ instar until the end of pupation. The cessation of peptide translation is fatal to living organisms, and PGE_2_ is necessary for the maintenance of immunity in insects (39). Furthermore, *eIFs* and *PCCPs* dsRNA affect the development and immunity of armyworm in many ways. In the feeding stage, these three pathways coordinate to present a “no gap” coordination.

Inhibition of three other pathways involving *eIFs*, *Inxs*, and *PCCPs*, causes immunosuppression in residual surviving *S. frugiperda* larvae. Silencing of these three pathways decreased development and increased the degree of early apoptosis, both of which are hallmarks of immunosuppression by bracovirus. This is not surprising because the bracovirus inhibits host immunosuppression in *S. litura* (4, 40). However, hosts only infected by bracovirus without a parasitoid are not known. Here, our results fill the gaps in which a truly immunosuppressive function of bracovirus is noted in different larval stages and not just at the lower instar stage. In the feeding stage, these three pathways present a unit coordination towards immunosuppression in residual *S. frugiperda* larvae.

*eIFs*, *Inxs*, and *PCCP* dsRNAs co-operatively function against *S. frugiperda* in its non-feeding stage; their dsRNA silence four pathways involving PCCPs, eIFs, Inxs, and Dip3, which undertake different functions against *S. frugiperda*. It is well-known that the intricate signaling pathways are not independent of each other. The occurrence of tumors, formation of autophagosomes, activation of immune signals, damage and repair of DNA, and the process of aging are all completed through the cooperation of multiple signaling pathways (41, 42). Similar to the bracovirus, 14 *Mix-T* dsRNA worked together to block the immune pathway in the larvae of armyworm and eventually led to immunosuppression throughout the life cycle. Only *PCCPs* dsRNA can kill the non-feeding pupal stage. Blocking humoral immunity reduced the antimicrobial peptide expression. This is because the absence of antimicrobial peptides is fatal to an insect’s innate immunity (43). *PCCPs* dsRNA specifically regulates the synthesis of PGE_2_ and thus reduces the production of antimicrobial peptides. *Inxs* and *eIFs* dsRNA increased the time of development of the residual surviving larvae. Increased time for the completion of life cycle leads to reduced generations. It is yet unclear how *eIFs* and *Inxs* dsRNA increase the duration of the pupal stage. Recently, it has been reported that the use of triazole can effectively alter the timing of metamorphosis of digger wasps (44). Triazole achieves insecticidal effect mainly by inhibiting enzyme activity in insects, which is quite different from the role of *eIFs* dsRNA. *eIFs* dsRNA inhibits the synthesis of many major proteins and ultimately affects the life cycle of armyworm. Similar to the altered life-cycle of crabronid wasp due to the blocking of the neurotransmitter acetylcholine by acetamiprid (45), the blocking of small molecules transmission between insect cells by *Inxs* dsRNA affects the communication between cells and ultimately alters the life-cycle of armyworm.

Regarding the cost and practically feasibility of dsRNA, the current cost of controlling pests through dsRNA technology is undeniably higher than that of pesticides for weed management in turfgrass systems (46). Spraying dsRNA has been used to control the western flower thrips in greenhouse (36). We believe with advances in research and the maturation of technology, the cost will be reduced, and the use of dsRNA could become practically feasible.

In summary, we have demonstrated that simulating bracovirus by dsRNA provides new insights into understanding the coordination among natural enemies. In addition, our results reveal a truly immunosuppressive function of bracovirus, thereby allowing the avoidance of the self-protection strategy from parasitoids, which are negative factors in biocontrol. Our findings provide a new perspective on bracovirus–parasitoid–host interactions.

## Data availability statement

The original contributions presented in the study are included in the article/**Supplementary Material**. Further inquiries can be directed to the corresponding author.

## Author contributions

XL: Data curation, Investigation, Methodology, Writing – original draft, Writing – review & editing. YM: Investigation, Methodology, Writing – original draft, Writing – review & editing. JL: Investigation, Methodology, Writing – original draft, Writing – review & editing. XY: Investigation, Methodology, Writing – original draft, Writing – review & editing. NP: Investigation, Methodology, Writing – original draft, Writing – review & editing. CC: Investigation, Methodology, Writing – original draft, Writing – review & editing. WZ: Investigation, Methodology, Writing – original draft, Writing – review & editing. YH: Methodology, Writing – original draft, Investigation. XQ: Investigation, Methodology, Writing – original draft. LZ: Investigation, Methodology, Writing – original draft. QC: Investigation, Methodology, Writing – original draft, Writing – review & editing. CC: Investigation, Methodology, Writing – original draft. GZ: Investigation, Methodology, Writing – original draft. YH: Investigation, Methodology, Writing – original draft. HL: Investigation, Methodology, Writing – original draft. QZ: Investigation, Methodology, Writing – original draft. HT: Investigation, Methodology, Writing – original draft. JM: Investigation, Methodology, Writing – original draft. KL: Conceptualization, Data curation, Funding acquisition, Investigation, Methodology, Software, Supervision, Writing – original draft, Writing – review & editing.

## References

[1] Cui JH Dong SM Chen CX Xiao W Cai QC Zhang LD. Microplitis bicoloratus bracovirus modulates innate immune suppression through the eIF4E-eIF4A axis in the insect Spodoptera litura. Dev Comp Immunol (2019) 95:101–7. doi: 10.1016/j.dci.2019.02.010 30776419

[2] Zhang LD Cai QC Cui JH Zhang W Dong SM Xiao W. A secreted-Cu/Zn superoxide dismutase from Microplitis bicoloratus reduces reactive oxygen species triggered by symbiotic bracovirus. Dev Comp Immunol (2019) 92:129–39. doi: 10.1016/j.dci.2018.11.014 30471301

[3] Zhou GF Chen CX Cai QC Yan X Peng NN Li XC. Bracovirus sneaks into apoptotic bodies transmitting immunosuppressive signaling driven by integration-mediated eIF5A hypusination. Front Immunol (2022) 13:901593. doi: 10.3389/fimmu.2022.901593 35664011 PMC9156803

[4] Chen CX He HJ Cai QC Zhang W Kou TC Zhang XW. Bracovirus-mediated innexin hemichannel closure in cell disassembly. iScience (2021) 24(4):102281. doi: 10.1016/j.isci.2021.102281 33817584 PMC8008186

[5] Cai QC Chen CX Liu HY Zhang W Han YF Zhang Q. Interactions of Vank proteins from Microplitis bicoloratus bracovirus with host Dip3 suppress eIF4E expression. Dev Comp Immunol (2021) 118:103994. doi: 10.1016/j.dci.2021.103994 33417999

[6] Keerthiraju E Du C Tucker G Greetham D. A role for COX20 in tolerance to oxidative stress and programmed cell death in saccharomyces cerevisiae. Microorganisms (2019) 7(11):1–12. doi: 10.3390/microorganisms7110575 PMC6920987 31752220

[7] Luo K Trumble JT Pang Y. Development of Microplitis bicoloratus on *Spodoptera litura* and implications for biological control. BioControl (2006) 52(3):309–21. doi: 10.1007/s10526-006-9030-8

[8] Goergen G Kumar PL Sankung SB Togola A Tamo M. First report of outbreaks of the fall armyworm spodoptera frugiperda (J E smith) (Lepidoptera, noctuidae), a new alien invasive pest in west and central africa. PloS One (2016) 11(10):e0165632. doi: 10.1371/journal.pone.0165632 27788251 PMC5082806

[9] Harrison RD Thierfelder C Baudron F Chinwada P Midega C Schaffner U. Agro-ecological options for fall armyworm (Spodoptera frugiperda JE Smith) management: Providing low-cost, smallholder friendly solutions to an invasive pest. J Environ Manage (2019) 243:318–30. doi: 10.1016/j.jenvman.2019.05.011 31102899

[10] Wang W He P Zhang Y Liu T Jing X Zhang S. The population growth of spodoptera frugiperda on six cash crop species and implications for its occurrence and damage potential in China. Insects (2020) 11(9):1–14. doi: 10.3390/insects11090639 PMC7563954 32957580

[11] Silver A. Caterpillar’s devastating march across China spurs hunt for native predator. Nature (2019) 570(7761):286–7. doi: 10.1038/d41586-019-01867-3 31213690

[12] Tay WT Rane RV Padovan A Walsh TK Elfekih S Downes S. Global population genomic signature of Spodoptera frugiperda (fall armyworm) supports complex introduction events across the Old World. Commun Biol (2022) 5(1):297. doi: 10.1038/s42003-022-03230-1 35393491 PMC8989990

[13] Cheng T Wu J Wu Y Chilukuri RV Huang L Yamamoto K. Genomic adaptation to polyphagy and insecticides in a major East Asian noctuid pest. Nat Eco Evol (2017) 2017(1):1747–56. doi: 10.1038/s41559-017-0314-4 28963452

[14] Xiao H Ye X Xu H Mei Y Yang Y Chen X. The genetic adaptations of fall armyworm Spodoptera frugiperda facilitated its rapid global dispersal and invasion. Mol Ecol Resour (2020) 20(4):1050–68. doi: 10.1111/1755-0998.13182 32359007

[15] Nandakumar S Ma H Khan AS. Whole-genome sequence of the spodoptera frugiperda sf9 insect cell line. Genome Announc (2017) 5(34):1–2. doi: 10.1128/genomeA.00829-17 PMC5571409 28839023

[16] Li M Pang Z Xiao W Liu X Zhang Y Yu D. A transcriptome analysis suggests apoptosis-related signaling pathways in hemocytes of Spodoptera litura after parasitization by Microplitis bicoloratus. PloS One (2014) 9(10):e110967. doi: 10.1371/journal.pone.0110967 25350281 PMC4211697

[17] Zotti MJ Smagghe G. RNAi technology for insect management and protection of beneficial insects from diseases: lessons, challenges and risk assessments. Neotrop Entomol (2015) 44(3):197–213. doi: 10.1007/s13744-015-0291-8 26013264

[18] Zhang J Khan SA Hasse C Ruf S Heckel DG Bock R. Pest control. Full crop protection from an insect pest by expression of long double-stranded RNAs in plastids. Science (2015) 347(6225):991–4. doi: 10.1126/science.1261680 25722411

[19] Liu S Jaouannet M Dempsey DA Imani J Coustau C Kogel KH. RNA-based technologies for insect control in plant production. Biotechnol Adv (2020) 39:107463. doi: 10.1016/j.bioteChadv.2019.107463 31678220

[20] Wytinck N Manchur CL Li VH Whyard S Belmonte MF. dsRNA uptake in plant pests and pathogens: insights into RNAi-based insect and fungal control technology. Plants (Basel) (2020) 9(12):1–17. doi: 10.3390/plants9121780 PMC7765514 33339102

[21] Yoon JS Gurusamy D Palli SR. Accumulation of dsRNA in endosomes contributes to inefficient RNA interference in the fall armyworm, Spodoptera frugiperda. Insect Biochem Mol Biol (2017) 90:53–60. doi: 10.1016/j.ibmb.2017.09.011 28951282

[22] Terenius O Papanicolaou A Garbutt JS Eleftherianos I Huvenne H Kanginakudru S. RNA interference in Lepidoptera: an overview of successful and unsuccessful studies and implications for experimental design. J Insect Physiol (2011) 57(2):231–45. doi: 10.1016/j.jinsphys.2010.11.006 21078327

[23] Dalaisón-Fuentes LI Pascual A Crespo M Andrada NL Welchen E Catalano MI. Knockdown of double-stranded RNases (dsRNases) enhances oral RNA interference (RNAi) in the corn leafhopper, Dalbulus maidis. Pesticide Biochem Physiol (2023) 196:1–11. doi: 10.1016/j.pestbp.2023.105618 37945254

[24] Wang XZ Chen JS Wang W Niu DB Wu HZ Palli SR. Knockdown of the glutamate-gated chloride channel gene decreases emamectin benzoate susceptibility in the fall armyworm, Spodoptera frugiperda. Pestic Biochem Physiol (2023) 196:105636. doi: 10.1016/j.pestbp.2023.105636 37945267

[25] Saleh MC van Rij RP Hekele A Gillis A Foley E O’Farrell PH. The endocytic pathway mediates cell entry of dsRNA to induce RNAi silencing. Nat Cell Biol (2006) 8(8):793–U19. doi: 10.1038/ncb1439 16862146 PMC2731564

[26] Rocha KL Mangine T Harris EJ Lawrence PO. Immature stages of Fopius arisanus (Hymenoptera: Braconidae) in Bactrocera dorsalis (Diptera: Tephritidae). Florida Entomol (2004) 87(2):164–8. doi: 10.1653/0015-4040(2004)087[0164:Isofah]2.0.Co;2

[27] Oleson BJ Bazopoulou D Jakob U. Shaping longevity early in life: developmental ROS and H3K4me3 set the clock. Cell Cycle (2021) 20(22):2337–47. doi: 10.1080/15384101.2021.1986317 PMC8794500 34657571

[28] Luo K Pang Y. Spodoptera litura multicapsid nucleopolyhedrovirus inhibits Microplitis bicoloratus polydnavirus-induced host granulocytes apoptosis. J Insect Physiol (2006) 52(8):795–806. doi: 10.1016/j.jinsphys.2006.04.007 16764883

[29] Bazopoulou D Knoefler D Zheng Y Ulrich K Oleson BJ Xie L. Developmental ROS individualizes organismal stress resistance and lifespan. Nature (2019) 576(7786):301–5. doi: 10.1038/s41586-019-1814-y PMC7039399 31801997

[30] Giancaspero TA Dipalo E Miccolis A Boles E Caselle M Barile M. Alteration of ROS homeostasis and decreased lifespan in S. cerevisiae elicited by deletion of the mitochondrial translocator FLX1. BioMed Res Int (2014) 2014:101286. doi: 10.1155/2014/101286 24895546 PMC4033422

[31] Lagnado A Leslie J Ruchaud-Sparagano MH Victorelli S Hirsova P Ogrodnik M. Neutrophils induce paracrine telomere dysfunction and senescence in ROS-dependent manner. EMBO J (2021) 40(9):e106048. doi: 10.15252/embj.2020106048 33764576 PMC8090854

[32] Parkinson RH Gray JR. Neural conduction, visual motion detection, and insect flight behaviour are disrupted by low doses of imidacloprid and its metabolites. Neurotoxicology (2019) 72:107–13. doi: 10.1016/j.neuro.2019.02.012 30790592

[33] Branan N. Neuron cannibalism: hungry male cells consume their own proteins. Sci Am Mind (2009) 20(4):9–9. doi: 10.1038/scientificamericanmind0709-9a

[34] Zhang JJ Sun LL Wang YN Xie GY An SH Chen WB. and zhao XC: serotonergic neurons in the brain and gnathal ganglion of larval spodoptera frugiperda. Front Neuroanat (2022) 16:844171. doi: 10.3389/fnana.2022.844171 35360650 PMC8960143

[35] Cooper AM Silver K Zhang JZ Park Y Zhu KY. Molecular mechanisms influencing efficiency of RNA interference in insects. Pest Manage Sci (2019) 75(1):18–28. doi: 10.1002/ps.5126 29931761

[36] Khan F Kim M Kim Y. Greenhouse test of spraying dsRNA to control the western flower thrips, infesting hot peppers. BMC Biotechnol (2023) 23(1):1–12. doi: 10.1186/s12896-023-00780-y PMC10074877 37016358

[37] Li SC Kim DS Zhang J. Plastid-mediated RNA interference: A potential strategy for efficient pest control. Plant Cell Environ (2023) 46(9):2595–605. doi: 10.1111/pce.14652 37332196

[38] Zhu KY Palli SR. Mechanisms, applications, and challenges of insect RNA interference. Annu Rev Entomol (2020) 65:293–311. doi: 10.1146/annurev-ento-011019-025224 31610134 PMC9939233

[39] Ahmed S Kim Y. Prostaglandin catabolism in Spodoptera exigua, a lepidopteran insect. J Exp Biol (2020) 223(Pt 21):1–10. doi: 10.1242/jeb.233221 32978320

[40] Dong SM Cui JH Zhang W Zhang XW Kou TC Cai QC. Inhibition of translation initiation factor eIF4A is required for apoptosis mediated by Microplitis bicoloratus bracovirus. Arch Insect Biochem Physiol (2017) 96(3):e21423. doi: 10.1002/arch.21423 28940716

[41] Hoesel B Schmid JA. The complexity of NF-κB signaling in inflammation and cancer. Mol Cancer (2013) 12:86. doi: 10.1186/1476-4598-12-86 23915189 PMC3750319

[42] Zhou C Liang Y Zhou L Yan Y Liu N Zhang R. TSPAN1 promotes autophagy flux and mediates cooperation between WNT-CTNNB1 signaling and autophagy via the MIR454-FAM83A-TSPAN1 axis in pancreatic cancer. Autophagy (2021) 17(10):3175–95. doi: 10.1080/15548627.2020.1826689 PMC8525961 32972302

[43] Eleftherianos I Zhang W Heryanto C Mohamed A Contreras G Tettamanti G. Diversity of insect antimicrobial peptides and proteins - A functional perspective: A review. Int J Biol Macromol (2021) 191:277–87. doi: 10.1016/j.ijbiomac.2021.09.082 34543628

[44] Heneberg P Bogusch P. Commonly used triazole fungicides accelerate the metamorphosis of digger wasps (Hymenoptera: Spheciformes). Environ Sci pollut Res Int (2022) 29(44):67430–41. doi: 10.1007/s11356-022-22684-8 36029446

[45] Heneberg P Bogusch P Astapenková A Řezáč M. Neonicotinoid insecticides hinder the pupation and metamorphosis into adults in a crabronid wasp. Sci Rep (2020) 10(1):7077. doi: 10.1038/s41598-020-63958-w 32341495 PMC7184726

[46] Ethridge SR Grieger K Locke AM Everman WJ Jordan DL Leon RG. Views of RNAi approaches for weed management in turfgrass systems. Weed Sci (2023) 71(4):344–52. doi: 10.1017/wsc.2023.37

