# Supplementary material for: Simulating immunosuppressive mechanism of Microplitis bicoloratus bracovirus coordinately fights Spodoptera frugiperda
Source: Front Immunol. 2023 Dec 11;14:1289477. doi: 10.3389/fimmu.2023.1289477 (PMC10749342; doi:10.3389/fimmu.2023.1289477)
Supplement: Supplementary file 1 [file DataSheet_1.docx]

Supplementary Materials for

**Simulating immunosuppression against *Microplitis bicoloratus bracovirus* coordinately fights *Spodoptera frugiperda***

Xing-Cheng Li†, Yin-Chen Ma†, Jin Long†, Xiang Yan†, Nan-Nan Peng†, Cheng-Hui Cai†, Wen-Feng Zhong†, Yong-Biao Huang, Xin Qiao, Xiang-Li Zhou, Qiu-Chen Cai, Chang-Xu Cheng, Gui-Fang Zhou, Yun-Feng Han, Hong-Yu Liu, Qi Zhang, Hong-Mei Tang, Jiang-Hui Meng, Kai-Jun Luo*

*Corresponding author. Email: kaijun_luo@ynu.edu.cn

†These authors contributed equally to this work

This file includes:

Materials and Methods

Figs. S1 to S4

References

**Materials and Methods**

**Insects**

The *Spodoptera frugiperda* colonies were grown on an artificial diet (1) at 27 ± 1 °C and 60%–80% humidity until the larvae reached the 2^nd^ instar. They were then separated and individually reared in a 25 mL container (height, 2.8 cm; top diameter, 4 cm; bottom diameter, 3 cm), until the end of pupation and eclosion. Adults were provided with honey as their dietary supplement.

**Plasmid construction for dsRNA preparation**

Plasmids were constructed as described previously (2). First, we compared the sequence homology of *Spodoptera litura* with *S. frugiperda* sequences to identify conserved sequence fragments for subsequent dsRNA analysis and design that are targeted by bracovirus, a symbiont of the wasp *Microplitis bicoloratus*. The sequences encoding *eIF4G*, *COX11*, *COX20*, *PGES*, *PLA_2_*, *ecSOD49*, *ecSOD58*, or *ecSOD67* were inserted into the RNAi vector L4440, containing two convergent T7 polymerase promoters in opposite orientation, separated by a multiple cloning site. The sequenced plasmids for dsRNA feeding were transformed into the bacterial host *E. coli* HT115 (DE3). *Dip3,* *eIF4A*, *eIF4E*, *eIF5A*, *DHYS*, *DOHH*, *Inx1*, *Inx2*, *Inx3*, and *Inx4* stocks were used as described previously(3-7). Four stocks, *PLA_2_*, *COX11*, *COX20*, and *PGES* were used for the preparation of the mixture *PCCPs* dsRNA, four stocks, *Inx1*, *Inx2*, *Inx3*, and *Inx4*, for mixture *Inxs* dsRNA, six stocks, *eIF4G*, *eIF4A*, *eIF4E*, *eIF5A*, for mixture *eIFs* dsRNA, one stock, *Dip3*, for *Dip3* dsRNA, and three stocks*, M. bicoloratus ecSOD49*, *ecSOD58*, and *ecSOD67*, for the preparation of mixture *SODS* dsRNA. In total, 14 stocks (*PGES, Dip3,* *eIF4A*, *eIF4E*, *eIF5A*, *DHYS*, *DOHH*, *Inx1*, *Inx2*, *Inx3*, and *Inx4*) were used for the preparation of the *Mix-T* dsRNAs.

**Preparation of dsRNAs and dsRNA mixtures in the artificial diet and dsRNA mixtures liquids**

The preparation of dsRNAs was performed according to previously published protocols (3, 6). Briefly, a single colony of HT115 containing the recombinant L4440 vector was inoculated in 4 mL LB medium containing 4 μL ampicillin (100 μg/mL) and 4 μL tetracycline (100 μg/mL), and cultured overnight at 37 °C. The expression strain was cultured until it reached an OD_595_ of 0.6 and IPTG (working solution concentration 0.4 mmol/L) was added for 4–5 h of induction. The recombinant strain was centrifuged at 10,000 × *g* for 20 min, the supernatant was discarded, and the precipitate was collected. For the 14 dsRNA mixture, 14 precipitates were resuspended with ddH_2_O individually, and later mixed together.

To obtain dsRNA mixtures in the artificial diet(6), bacterial precipitates with dsRNA targeting the protein translation pathway, *eIF4A*, *eIF4E*, *eIF4G*, *eIF5A*, *DHYS*, and *DOHH* (*eIFs*) were mixed; for the humoral immunity pathway, *PGES*, *COX11*, *COX20*, and *PLA_2_* (*PCCPs*) were mixed; for cell communication pathways, *Inx1*, *Inx2*, *Inx3* and *Inx4* (*Inxs*) bacterial precipitates were mixed; for ROS signaling pathway *M. bicoloratus* *ecSOD49*, *58*, *67* (*SODs* dsRNA) bacterial precipitates were mixed; for the gene transcription pathway, only *Dip3* bacterial precipitates was used. Finally, bacterial precipitates were mixed with 50 mL of freshly prepared artificial diet after it was cooled to 50°C. The prepared dsRNA feeding diet was stored at 4^o^C and as feed in a small plastic box (9.5 × 7 × 5.5 cm^3^, 200 mL) up to 1 week. Before hatching, the eggs were placed on artificial diet. Larvae were provided with fresh food every day.

To obtain *Mix-T* dsRNA mixture liquids, the suspension was ultrasonicated in an ice water bath at 150 W for 30 min until it cleared. Following centrifugation, the supernatant containing various other molecules, along with dsRNAs was collected and the mixtures were diluted to the corresponding concentrations. The dsRNA in the bacterial lysates was stored at -80°C until further use without further purification.

**One-time or continuous feeding of mixed dsRNAs to *S. frugiperda***

For the one-time feeding of the 14 *Mix-T* dsRNAs, the mixtures of the 14 dsRNAs were dropped onto the artificial diet only once, following which a fresh regular artificial diet without dsRNAs was used for the remaining experimental days. For the continuous feeding of the 14 *Mix-T* dsRNAs, the mixtures of the 14 dsRNAs were dropped onto the artificial diet, following which a new diet containing the dsRNA mixture was used for the remaining experimental days. *Mix-T no SODs* dsRNA, mixed *eIFs* dsRNA, mixed *PCCPs* dsRNA, mixed *Inxs* dsRNA, mixed *SODs* dsRNA and *Dip3* dsRNA were used for continuous feeding. *egfp* dsRNA and ddH_2_O were used as controls. Fifty larvae were selected for each treatment group; the number of surviving larvae was counted every day until pupation ended. The surviving pupae were counted until all of them had eclosed. The head capsule width was captured in images every day using an EZcam of binocular microscope (SZX-ZB7, Olympus, Japan) and was measured using the EZcam Ver.5.0 software (OPLENIC, Beijing, China).

**Colony feeding and individual feeding dsRNAs**

In the 14 *Mix-T* dsRNA one-time feeding assays, based on the behavior of *S. frugiperda* in the field, we designed assays for larvae in colonies from 1^st^ to 2^nd^ and for individual larvae from 3^rd^ to 6^th^ instars, using continuous and one-time feeding methods. In the colony cannibalism assays, colonies from 1^st^ to 2^nd^ instar were used, without separating the individual larvae. In the individual assays, larvae were separated from the 1^st^ instar onwards.

**qRT-PCR for the detection of the mRNAs of *S. frugiperda* genes after mixed dsRNA feeding**

qRT-PCR analyses were performed according to the previously published protocols (4, 7). Briefly, after nine days of RNAi feeding, hemocytes were extracted from *S. frugiperda*. The 18S rDNA gene was used as a housekeeping gene for normalization. Each sample was tested in triplicates and the 2^-ΔΔCT^ method was used, as previously described(8).

**Analysis of hemocyte apoptosis using flow cytometry**

Apoptosis was tested using the Annexin V-FITC/PI apoptosis detection kit, as described previously (3, 9). Briefly, after nine days of RNAi feeding, hemocytes (2×10^5^) were extracted from *S. frugiperda* (5∼10 larvae), resuspended in 195 μL of 1× binding buffer, and incubated with 5 μL Annexin V-FITC dye and 2.5 μL PI dye was used for 15 min in the dark. Dual-color flow cytometry was performed. Approximately 10,000 cells were counted in each sample, and each sample was tested in triplicate.

**Western blotting of hemocytes**

Western blotting was performed as described previously (10). Briefly, hemocytes were extracted from *S. frugiperda* after 9 days of feeding with mixed dsRNAs and the protein concentrations were measured using a BCA protein quantification kit. Samples (15 μg) were separated using SDS-polyacrylamide gel electrophoresis and transferred onto PVDF membranes. After incubation with antibodies, the bands were visualized using enhanced chemiluminescence (Beyotime).

**Dye uptake and ROS detection.**

Dye uptake analysis (7) and ROS detection (11) were performed as described previously using an ROS detection kit. Briefly, after nine days of RNAi feeding, hemocytes were extracted from *S. frugiperda*. A total of 1×10^5^ hemocytes were incubated with TO-PRO-3 for 15 min in the dark for dye uptake detection. The same number of hemocytes were incubated with 0.1 μL DCFH-DA for 30 min at 37°C in the dark for ROS detection. For detection of both, five images were recorded per plate using the 20× objective lens (both bright and fluorescent fields) of an inverted fluorescence microscope (Olympus 1X71) at room temperature. Image J was used to measure the fluorescence intensity of the images.

**Spraying of 14 *Mix-T* dsRNAs in the field**

The corn field was divided into several quadrants with a length x width of 2 x 2 square meters each quadrat containing about 50 corn plants. The dsRNA mixtures were diluted to concentrations of 100 ng/μL, 250 ng/μL and 500 ng/μL and sprayed onto the surface of corn leaves at 1 L/square. The control treatment consisted of spraying ddH_2_O or *egfp* dsRNA. The *S. frugiperda* larvae were randomly placed into each corn plant sample. Each sample consisted of 1^st^, 2^nd^, and 3^rd^ instar larvae: 15–20 1^st^, 15–20 2^nd^, 15–20 3^rd^ instar larvae and one egg piece (about 50 eggs). Five days later, the disaster situation of corn was assessed, and the degree of damage of the corn plants was statistically analyzed according to the Davis survey method.

**Statistical analysis**

Survival cure analysis were compared using the log-rank (Mantel–Cox) test. The survival larvae at the end of pupation and survival pupae at the end of eclosion were compared using unpaired Student’s *t*-test with Holm–Sidak method for multiple *t* test. Head capsule width of residual survival larvae was analyzed by two-way ANOVA. The head capsule width was compared using the Tukey’s multiple comparisons test. Data were analyzed using GraphPad Prism (ver. 7, Prism), and statistical significance was determined using the Student’s *t*-test for unpaired experiments (two-tailed). Statistical significance was set to *p** < 0.05. The resulting data are presented as the means ± SEM from at least three independent experiments.

**Supplementary Figures**

**
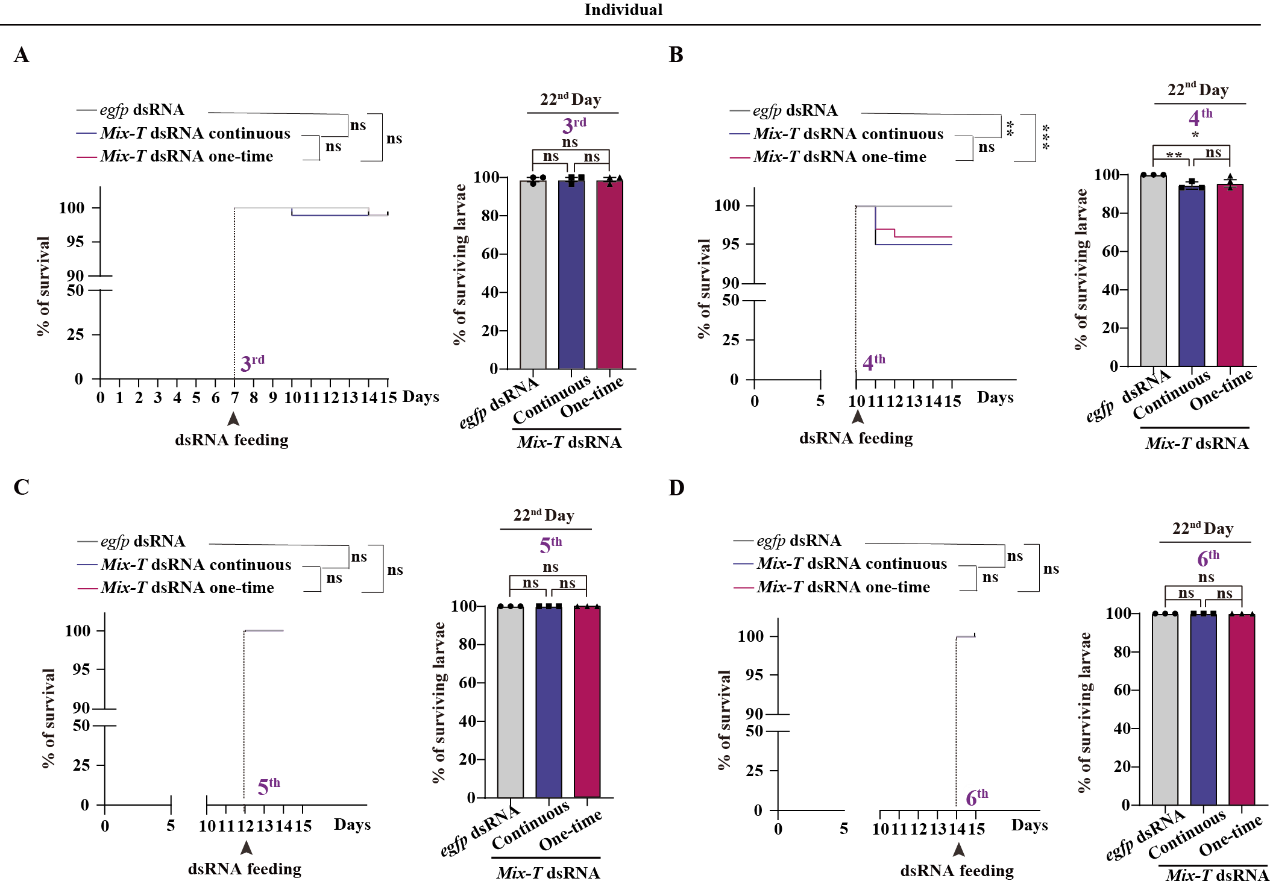
**

**Supplementary Fig. 1. Simulating bracoviral attack on *S. frugiperda* via the one-time feeding of *Mix-T* dsRNA (related to Fig. 1).**

**(A)** Survival curve of individual 3^rd^ instar *S. frugiperda* larvae after feeding with *Mix-T* dsRNA from the first larval day to the 22^nd^ and the survival rate on the 22^nd^ day at the end of pupation.

ns, *p* (*egfp* dsRNA: *Mix-T* dsRNA continuous) = 0.9968; ns, *p* (*egfp* dsRNA: *Mix-T* dsRNA one-time) > 0.9999; ns, *p* (*Mix-T* dsRNA continuous: *Mix-T* dsRNA one-time) = 0.9968.

**(B)** Survival curve of individual 4^th^ instar *S. frugiperda* larvae after feeding with *Mix-T* dsRNA from the first larval day to the 22^nd^ and the survival rate on the 22^nd^ day at the end of pupation.

** *p* (*egfp* dsRNA: *Mix-T* dsRNA continuous) = 0.0237; *** *p* (*egfp* dsRNA: *Mix-T* dsRNA one-time) = 0.0437; ns, *p* (*Mix-T* dsRNA continuous: *Mix-T* dsRNA one-time) = 0.7341.

**(C)** Survival curve of individual 5^th^ instar *S. frugiperda* larvae after feeding with *Mix-T* dsRNA from the first larval day to the 22^nd^ and the survival rate on the 22^nd^ day at the end of pupation.

ns, *p* (*egfp* dsRNA: *Mix-T* dsRNA continuous) > 0.9999; ns, *p* (*egfp* dsRNA: *Mix-T* dsRNA one-time) > 0.9999; ns, *p* (*Mix-T* dsRNA continuous: *Mix-T* dsRNA one-time) > 0.9999.

**(D)** Survival curve of individual 6^th^ instar *S. frugiperda* larvae after feeding with *Mix-T* dsRNA from the first larval day to the 22^nd^ and the survival rate on the 22^nd^ day at the end of pupation.

ns, *p* (*egfp* dsRNA: *Mix-T* dsRNA continuous) > 0.9999; ns, *p* (*egfp* dsRNA: *Mix-T* dsRNA one-time) > 0.9999; ns, *p* (*Mix-T* dsRNA continuous: *Mix-T* dsRNA one-time) > 0.9999.

Survival curves were compared using the log-rank (Mantel–Cox) test [x^2^(1) = 3.595] in **(A)**, [x^2^(2) = 0.00003704] **in (B)**, [x^2^(2) = 4.805] **in (C)**, [x^2^(2) = N/A] in **(D)**, and [x^2^(2) = N/A] in **(E)**.

In all graphs, **p* < 0.05, ***p* < 0.01, ns, no significance; the error bars represent the SEM. Unpaired Student’s *t*-test with Holm–Sidak method for multiple *t* test; n = 3.

**
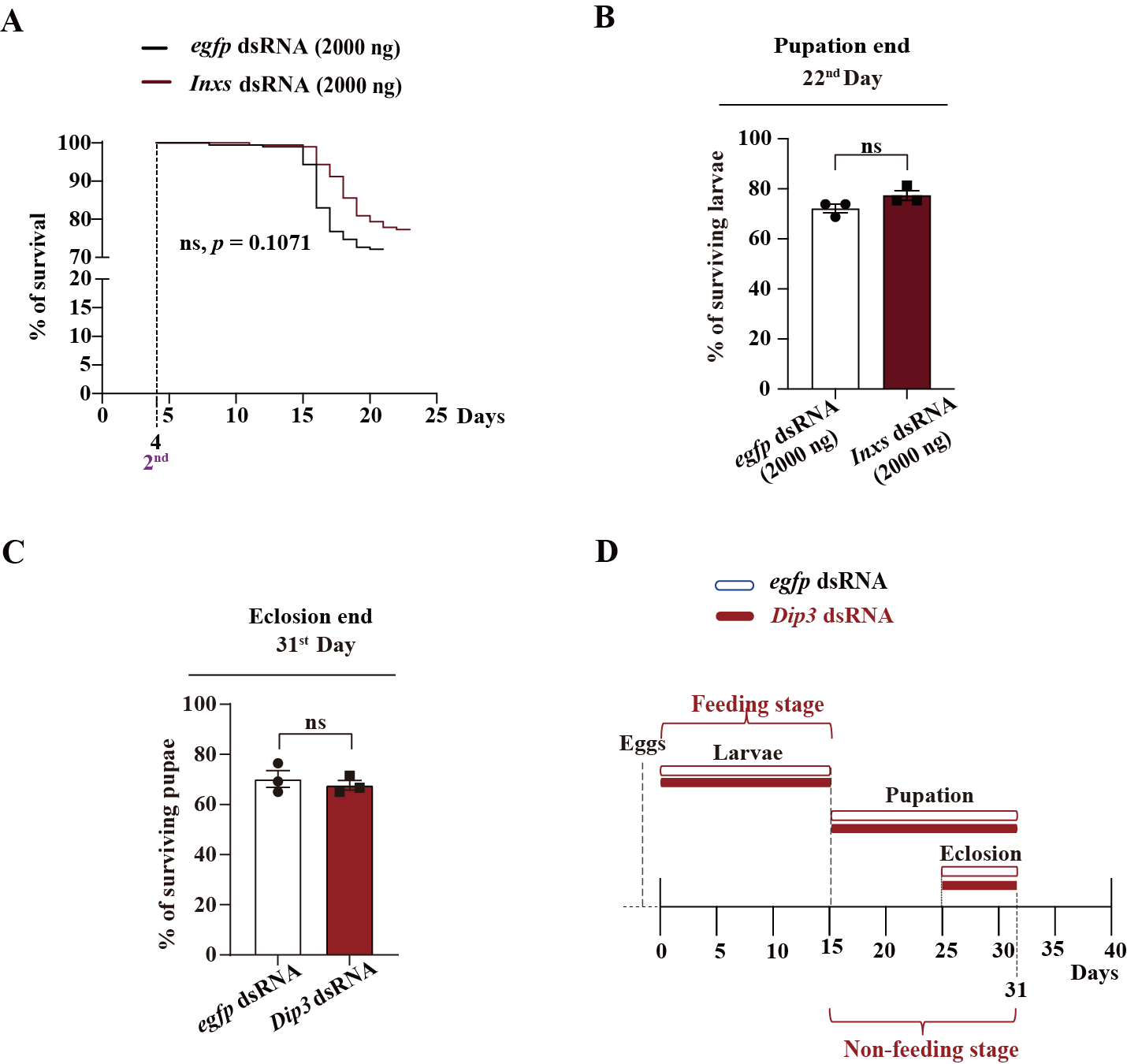
**

**Supplementary Fig. 2. *Dip3*, *eIFs*, *Inxs*, and *PCCPs* dsRNAs co-operatively function against *S. frugiperda* at the feeding and non-feeding stages (related to Fig. 3)**

**(A, B)** Survival curve of 2^nd^ instar *S. frugiperda* after feeding with the Mix-T dsRNA from the 4^th^ to the 22^nd^ day **(A)** and the survival rate at the 22^nd^ day (end of pupation) **(B)**.

**(C)** Surviving pupae of *S. frugiperda* after feeding with *Dip3* dsRNA (A) at the end of eclosion.

**(D)** Time of feeding and non-feeding (pupation and eclosion) stages of *S. frugiperda* after feeding with *Dip3* dsRNA.

Survival curves were compared by using the log-rank (Mantel–Cox) test [x^2^(1) = 2.596] in **(A)**.

In all graphs, ns, no significance; the error bars represent the SEM. Unpaired Student’s *t*-test with Holm–Sidak method for multiple *t* test; n = 3.

**
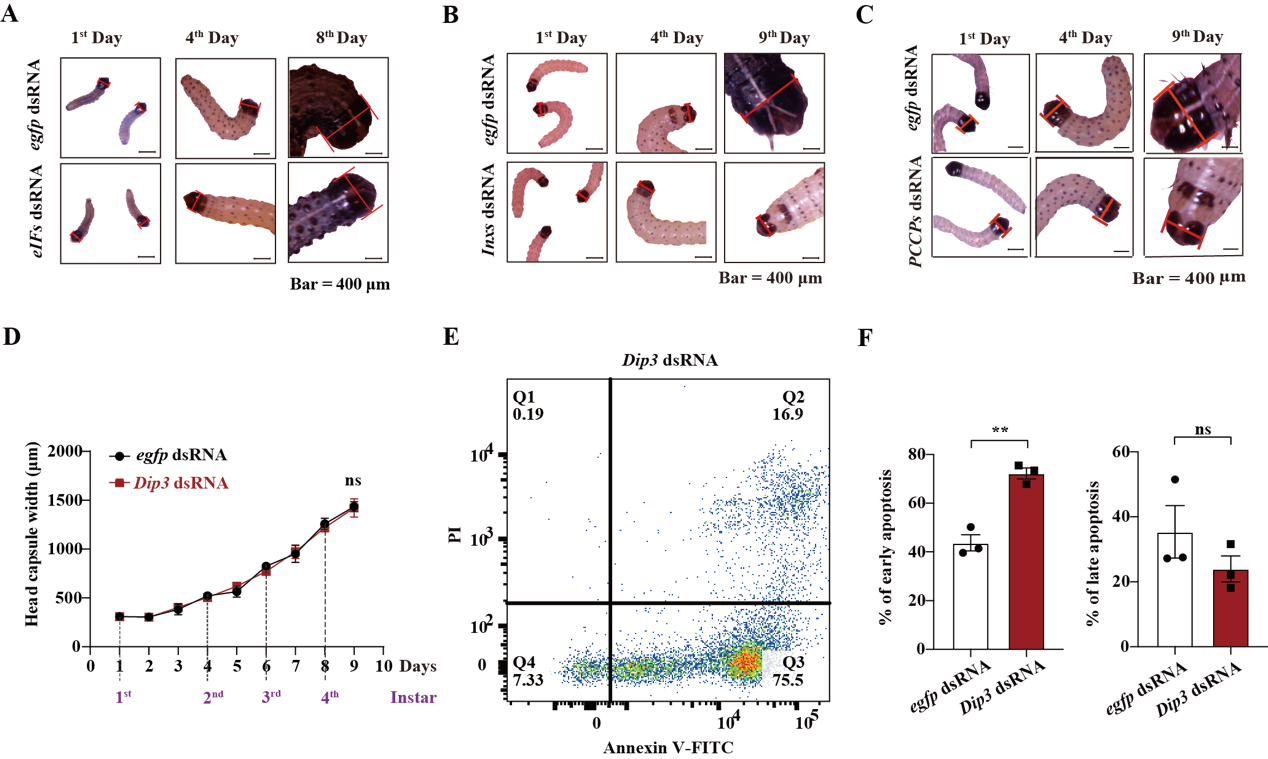
**

**Supplementary Fig. 3. *eIFs*, *Inxs*, and *PCCP* dsRNAs cause immunosuppression in the residual surviving *S. frugiperda* (related to Fig. 4).**

**(A–C)** Images of the head capsule of residual *S. frugiperda* larvae that survived, after feeding with *eIFs* (A), *Inxs* (B) and *PCCPs* (C) dsRNA individually at the 1^st^, 4^th^, and 9^th^ days. Scale bar, 400 µm.

**(D)** The head capsule width of residual surviving *S. frugiperda* larvae, after feeding with *Dip3* dsRNA individually within 1–9 days.

**(E, F)** Flow cytometry analysis of the apoptotic hemocytes of residual surviving *S. frugiperda* larvae, after feeding with *Dip3* dsRNA individually within 1–9 days.

In all graphs, ∗∗*p* < 0.01, ns, no significance; the error bars represent the SEM. Unpaired Student’s *t*-test with Holm–Sidak method for multiple *t* test; n = 3.

**
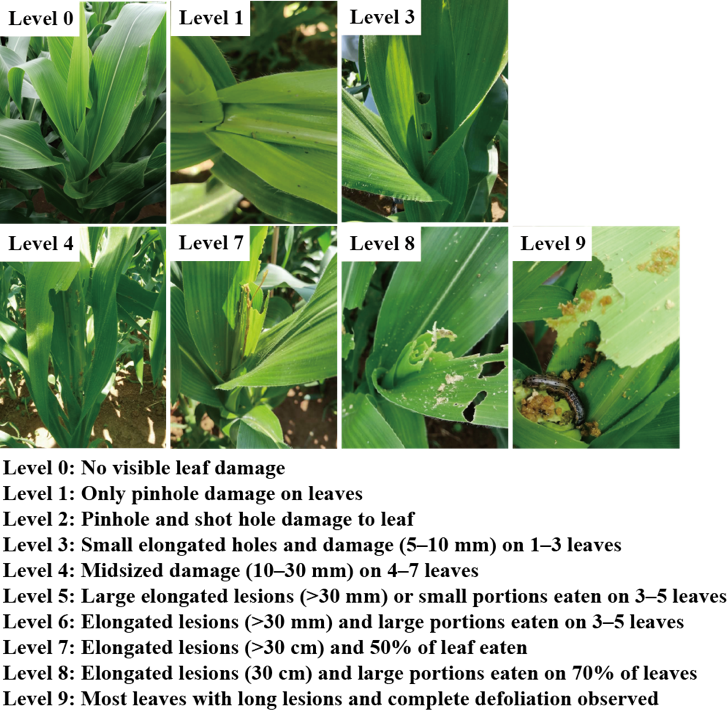
**

**Supplementary Fig. 4. *Mix-T* dsRNA can effectively control *S. frugiperda* in the field (related to Fig. 6).**

Visual rating scales for leaf damage assessment based on Davis method.

**Table S1.** Key resources list.

| **REAGENT or RESOURCE** | **SOURCE** | **IDENTIFIER** |
| --- | --- | --- |
| Antibodies | | |
| Mouse Monoclonal anti-GAPDH | Solarbio | Cat#M1000110; |
| Mouse anti-Tubulin | Transgen Biotech | Cat#HC101: RRID:AB_2893358 |
| Rabbit anti-CypA | GL Binchem synthesis | N/A |
| Rabbit anti-CypD | GL Binchem synthesis | N/A |
| Rabbit anti-eIF4A | ABclonal | Cat#A5294;RRID:AB_2766106 |
| Rabbit anti-eIF4E | ABclonal | Cat#A2162;RRID:AB_2764180 |
| Rabbit anti-eIF4G | ABclonal | Cat#A0881;RRID:AB_2757439 |
| Rabbit anti-eIF5A | ABclonal | Cat#A2016;RRID:AB_2764040 |
| Rabbit anti-p53 | ABclonal | Cat#A5761;RRID:AB_2766515 |
| Bacterial | | |
| DH5α | TaKaRa | Cat#9057 |
| HT115 | Self made | N/A |
| L4440 | MiaolingBio | Cat#P0311 |
| pBM16A | Biomed | Cat#CL071-01 |
| Chemicals, Peptides, and Recombinant Proteins | | |
| 5×Protein SDS-PAGE Loading Buffer | Beyotime | Cat#PO286 |
| Ampicillin,sodium salt | COOLABER | Cat#69-52-3 |
| IPTG | Sigma | Cat#I6758 |
| Native lysis Buffer | Solarbio | Cat#R0030 |
| PMSF | Solarbio | Cat#P0100 |
| RIPA buffer(high) | Solarbio | Cat#R0010 |
| Tetracycline HCL | BIOFROXX | Cat#1302GR005 |
| Zeocin Antibiotic | Thermo Fisher | Cat#R250 |
| Critical Commercial Assays | | |
| BCA protein quantitative kit | DINGGUO | Cat#BCA02 |
| Endo-free Plasmid Mini Kit II | omega | Cat#D6950-01B |
| Gel Extraction Kit | omega | Cat#D2500 |
| Plasmid Mini Kit I | omega | Cat#D6943-02 |
| ROS detection kit | JCBIO | Cat#E004-1-1 |
| Annexin V-FITC/PI apoptosis detection kit | Vazyme | Cat#A211-01 |
| RNAiso Plus kit | Takara Bio | Cat#9109 |
| Oligonucleotides | | |
| COX11 forward primer： AAGCTTATGATAGCTGTTGGAGTAATGACCG | This paper | N/A |
| COX11 reverse prime：  GGTACCCTTGACATATGAAGGTACAGGCAG | This paper | N/A |
| COX11-qPCR reverse primer：  ACAATCTCATCCACAAACTCCATCTT | This paper | N/A |
| COX11-qPCR forward primer： CAGTGACAGGCATCAGCACATACAA | This paper | N/A |
| COX20 forward primer： AAGCTTATGGATACAGTAGTTAAGA | This paper | N/A |
| COX20-qPCR forward primer： TTTCTGTATGGCATTTCAACTGGA | This paper | N/A |
| COX20-qPCR reverse primer：CCTTTCTGTTCAAGTTCTTTTTCG | This paper | N/A |
| COX2O reverse prime： GTCGACCTAAGCTGATTTTAATACA | This paper | N/A |
| DHYS-forward primer:  GAAGCTTGGGCAACTTATTAGTCCCAAA | Luo Lab stock | N/A |
| DHYS-reverse primer:  CCTCGAGGTTAAACATTCTTTTTATTGC | Luo Lab stock | N/A |
| dip3 forward primer：  GGATCCATGCAGTTCGAATA | Luo Lab stock | N/A |
| dip3 reverse prime：CTCGAGTTAAATCAACTTGTGTTC -3’ | Luo Lab stock | N/A |
| Dip3-qPCR forward primer： CAAAGACAAGCAAGCCACAGCC | This paper | N/A |
| Dip3-qPCR reverse primer：  CGCAGTACGTGCATCATATCCA | This paper | N/A |
| DOHH-forward primer:  GAAGCTTGACCTTAGAGAGTTACTAGAG | Luo Lab stock | N/A |
| DOHH-reverse primer:  CCTCGAGGTCAGCCCTCGACAGTAAGCA | Luo Lab stock | N/A |
| eIF4A forward primer：GATATCATGTCGTATTCACCAGA | Luo Lab stock | N/A |
| eIF4A reverse primer：TCTAGATAGATGAGGTTAGCCAC | Luo Lab stock | N/A |
| eIF4A-qPCR forward primer：GGTGGATTGGTTGACTGA | This paper | N/A |
| eIF4A-qPCR reverse primer：TGACGCATAATGACTTCG | This paper | N/A |
| eIF4E forward primer：GAGCTCATGGCTGGTAATAAT | Luo Lab stock | N/A |
| eIF4E reverse primer：GATATCCTAAACAGTGTACAGATTC | Luo Lab stock | N/A |
| eIF4E-qPCR forward primer：TCTGATTTGGACCGCTTCT | This paper | N/A |
| eIF4E-qPCR reverse primer：CGGCACCACAGATTTCG | This paper | N/A |
| eIF4G forward primer：CTCTAGAGATGAGTAGCAACGGGAATC | Luo Lab stock | N/A |
| eIF4G reverse primer：GGGTACCCAGTGGGCGCAGTGGGAGTT | Luo Lab stock | N/A |
| eIF4G-qPCR forward primer：AAGCGTTTGGAAGGTGT | This paper | N/A |
| eIF4G-qPCR reverse primer：TTGTCAGGCATCGTGGG | This paper | N/A |
| eIF5A forward primer：GAATTCATGGCTGATATCGAGGA | Luo Lab stock | N/A |
| eIF5A reverse prime：GCGGCCGCATTTGTCAA | Luo Lab stock | N/A |
| eIF5A-qPCR forward primer：TGCTTCAGTAACTTACCCTAT | This paper | N/A |
| eIF5A-qPCR reverse primer：CGTGTTTGCCAGTCTTTG | This paper | N/A |
| Inx1 forward primer：  GGATCCCAATAGGTTGCG | Luo Lab stock | N/A |
| Inx1 reverse primer：  CTCGAGATAAATATAGGTGAACAGC | Luo Lab stock | N/A |
| Inx1-qPCR forward primer：  GCGGTAGAGCGGACAC | Luo Lab stock | N/A |
| Inx1-qPCR reverse primer：  CGTGATGCGAGGGAATA | Luo Lab stock | N/A |
| Inx2 forward primer：GAATTCATGTTTGACGTTTTCGGCT | Luo Lab stock | N/A |
| Inx2 reverse primer：GCGGCCGCAACACACTGTCCTT | Luo Lab stock | N/A |
| Inx2-qPCR forward primer：  CGTTCCGTTTCTTTATCTG | Luo Lab stock | N/A |
| Inx2-qPCR reverse primer：  ACACGCTCCTCTGGCTC | Luo Lab stock | N/A |
| Inx3 forward primer： GAATTCATGGCGGTATTTGGTTTGG | Luo Lab stock | N/A |
| Inx3 reverse primer： GCGGCCGCAACGTTTCGGTTTC | Luo Lab stock | N/A |
| Inx3-qPCR forward primer：  ATCGCATCACATCAGCC | Luo Lab stock | N/A |
| Inx3-qPCR reverse primer：  AGGTAATCCAGCAATAGG | Luo Lab stock | N/A |
| Inx4 forward primer：  GGATCCATGTCAGATGCAGTC | Luo Lab stock | N/A |
| Inx4 reverse primer：  CTCGAGTATCTCTTGGTGACTCACT | Luo Lab stock | N/A |
| Inx4-qPCR forward primer：  AAGACGCCATCAACAGC | Luo Lab stock | N/A |
| Inx4-qPCR reverse primer：  GCCGAGCAGCACAAA | Luo Lab stock | N/A |
| PGES forward primer： GAGCTCATGCCGAAATAC | This paper | N/A |
| PGES reverse prime： ACTAGTGAATTCCGTAGGTCCTAC | This paper | N/A |
| PGES-qPCR forward primer： ATCAATCGCTCGCTACCTC | This paper | N/A |
| PGES-qPCR reverse primer：  ACAGCCTCGTCCTTTTCG | This paper | N/A |
| PLA_2_  reverse prime： GGTACCGAGCCACGTGCCTGGTTTATATAC | This paper | N/A |
| PLA_2_ forward primer： AAGCTTATGTCGTGCATTACATCTGTGCG | This paper | N/A |
| PLA_2_-qPCR forward primer： GAACCTGTGGAATGGCTGGAC | This paper | N/A |
| PLA_2_-qPCR reverse primer ：  TGTTGTTCGCCGTGTATGTAT | This paper | N/A |
| SOD49 forward primer：  TCTAGAATGAAATCAGTCATATTGC | This paper | N/A |
| SOD49 reverse prime：  GTCGACATAATTCCAATAACACCAC | This paper | N/A |
| SOD58 forward primer：  GGTACCATGAAATCAATCGTTTTA | This paper | N/A |
| SOD58 reverse prime：  CCGCGGGTTGTAATATTTGA | This paper | N/A |
| SOD67 forward primer：  GCTCTAGAATGAAATCAATCGTTATA | This paper | N/A |
| SOD67 reverse prime：  CCCAAGCTTTTAAGAGGATCCG | This paper | N/A |
| Recombinant DNA | | |
| L4440-COX11 | This paper | N/A |
| L4440-COX20 | This paper | N/A |
| L4440-DHYS | Luo Lab stock | N/A |
| L4440-Dip3 | Luo Lab stock | N/A |
| L4440-DOHH | Luo Lab stock | N/A |
| L4440-eGFP | Luo Lab stock | N/A |
| L4440-eIF4A | Luo Lab stock | N/A |
| L4440-eIF4E | Luo Lab stock | N/A |
| L4440-eIF4G | This paper | N/A |
| L4440-eIF5A | Luo Lab stock | N/A |
| L4440-Inx1 | Luo Lab stock | N/A |
| L4440-Inx2 | Luo Lab stock | N/A |
| L4440-Inx3 | Luo Lab stock | N/A |
| L4440-Inx4 | Luo Lab stock | N/A |
| L4440-PGES | This paper | N/A |
| L4440-PLA_2_ | This paper | N/A |
| L4440-SOD49 | This paper | N/A |
| L4440-SOD58 | This paper | N/A |
| L4440-SOD67 | This paper | N/A |
| Software and Algorithms | | |
| ApE | ApE | https://jorgensen.biology.utah.edu/wayned/ape/ |
| Prism8 | Graphpad | https://www.graphpad.com |
| Premier5 | Premie | N/A |
| ImageJ | ImageJ | https://imagej.nih.gov/ij/ |

**References**

1. Li G-H, Chen Q-J and Pang Y: Studies of artificial diets for the beet armyworm, Spodoptera exigua. *Acta Scientiae Circumstan- tiae/Huanjing Kexue Xuebao* (1998) 4: 1-5.

2. Timmons L, Court DL and Fire A: Ingestion of bacterially expressed dsRNAs can produce specific and potent genetic interference in *Caenorhabditis elegans*. *Gene* (2001) 263:(1-2) 103-12. doi:10.1016/s0378-1119(00)00579-5

3. Zhou GF, Chen CX, Cai QC, Yan X, Peng NN, Li XC, Cui JH, Han YF, Zhang Q, Meng JH, Tang HM, Cai CH, Long J and Luo KJ: Bracovirus Sneaks Into Apoptotic Bodies Transmitting Immunosuppressive Signaling Driven by Integration-Mediated eIF5A Hypusination. *Front Immunol* (2022) 13: 901593. doi:10.3389/fimmu.2022.901593

4. Cai QC, Chen CX, Liu HY, Zhang W, Han YF, Zhang Q, Zhou GF, Xu S, Liu T, Xiao W, Zhu QS and Luo KJ: Interactions of Vank proteins from Microplitis bicoloratus bracovirus with host Dip3 suppress eIF4E expression. *Dev Comp Immunol* (2021) 118: 103994. doi:10.1016/j.dci.2021.103994

5. Cui JH, Dong SM, Chen CX, Xiao W, Cai QC, Zhang LD, He HJ, Zhang W, Zhang XW, Liu T, Ding L, Yang Y, Lai JH, Zhu QS and Luo KJ: Microplitis bicoloratus bracovirus modulates innate immune suppression through the eIF4E-eIF4A axis in the insect Spodoptera litura. *Dev Comp Immunol* (2019) 95: 101-107. doi:10.1016/j.dci.2019.02.010

6. Dong SM, Cui JH, Zhang W, Zhang XW, Kou TC, Cai QC, Xu S, You S, Yu DS, Ding L, Lai JH, Li M and Luo KJ: Inhibition of translation initiation factor eIF4A is required for apoptosis mediated by *Microplitis bicoloratus* bracovirus. *Arch Insect Biochem Physiol* (2017) 96:(3) e21423. doi:10.1002/arch.21423

7. Chen CX, He HJ, Cai QC, Zhang W, Kou TC, Zhang XW, You S, Chen YB, Liu T, Xiao W, Zhu QS and Luo KJ: Bracovirus-mediated innexin hemichannel closure in cell disassembly. *iScience* (2021) 24:(4) 102281. doi:10.1016/j.isci.2021.102281

8. Livak KJ and Schmittgen TD: Analysis of relative gene expression data using real-time quantitative PCR and the 2−ΔΔCT method. *Methods* (2001) 25:(4) 402-8. doi:10.1006/meth.2001.1262

9. Luo K and Pang Y: Spodoptera litura multicapsid nucleopolyhedrovirus inhibits Microplitis bicoloratus polydnavirus-induced host granulocytes apoptosis. *J Insect Physiol* (2006) 52:(8) 795-806. doi:10.1016/j.jinsphys.2006.04.007

10. Liu T, Li M, Zhang Y, Pang Z, Xiao W, Yang Y and Luo K: A role for Innexin2 and Innexin3 proteins from Spodoptera litura in apoptosis. *PLoS One* (2013) 8:(7) e70456. doi:10.1371/journal.pone.0070456

11. Zhang LD, Cai QC, Cui JH, Zhang W, Dong SM, Xiao W, Li J, Kou TC, Zhang XW, He HJ, Ding L, Yang Y, Lai JH, Li M, Zhu QS and Luo KJ: A secreted-Cu/Zn superoxide dismutase from Microplitis bicoloratus reduces reactive oxygen species triggered by symbiotic bracovirus. *Dev Comp Immunol* (2019) 92: 129-139. doi:10.1016/j.dci.2018.11.014
